# Thyroid Hormone Transporters MCT8 and OATP1C1 Are Expressed in Pyramidal Neurons and Interneurons in the Adult Motor Cortex of Human and Macaque Brain

**DOI:** 10.3390/ijms24043207

**Published:** 2023-02-06

**Authors:** Yu Wang, Ting Wang, Ana Montero-Pedrazuela, Ana Guadaño-Ferraz, Estrella Rausell

**Affiliations:** 1 School of Medicine, Department of Anatomy Histology & Neuroscience, Autónoma de Madrid University (UAM), 28029 Madrid, Spain; 2 PhD Program in Neuroscience, Autónoma de Madrid University (UAM)-Cajal Institute, 28029 Madrid, Spain; 3 Instituto de Investigaciones Biomédicas Alberto Sols, Consejo Superior de Investigaciones Científicas (CSIC)-Autónoma de Madrid University (UAM), 28029 Madrid, Spain

**Keywords:** thyroid hormones, thyroid hormone transporters, MCT8, OATP1C1, primate, human, monkey, cerebral cortex, pyramidal cells, *Corpora amylacea*

## Abstract

Monocarboxylate transporter 8 (MCT8) and organic anion transporter polypeptide 1C1 (OATP1C1) are thyroid hormone (TH) transmembrane transporters that play an important role in the availability of TH for neural cells, allowing their proper development and function. It is important to define which cortical cellular subpopulations express those transporters to explain why MCT8 and OATP1C1 deficiency in humans leads to dramatic alterations in the motor system. By means of immunohistochemistry and double/multiple labeling immunofluorescence in adult human and monkey motor cortices, we demonstrate the presence of both transporters in long-projection pyramidal neurons and in several types of short-projection GABAergic interneurons in both species, suggesting a critical position of these transporters for modulating the efferent motor system. MCT8 is present at the neurovascular unit, but OATP1C1 is only present in some of the large vessels. Both transporters are expressed in astrocytes. OATP1C1 was unexpectedly found, only in the human motor cortex, inside the *Corpora amylacea* complexes, aggregates linked to substance evacuation towards the subpial system. On the basis of our findings, we propose an etiopathogenic model that emphasizes these transporters’ role in controlling excitatory/inhibitory motor cortex circuits in order to understand some of the severe motor disturbances observed in TH transporter deficiency syndromes.

## 1. Introduction

Thyroid hormones (TH), 3,5,3′-triiodothyronine (T3), and 3,5,3′,5′-tetraiodothyronine (thyroxine or T4) are indispensable for human brain development and functionality. In humans, the insufficiency or deficiency of TH signaling during development will lead to permanent and irreversible brain damage [1,2,3].

The main mechanism of TH action is the modulation of gene expression by the binding of the nuclear active hormone T3 to specific nuclear receptors, although indirect genomic actions by both T3 and T4 have also been described by their binding to different receptors at the cytoplasmic membrane, cytoplasm and mitochondria [4,5]. TH action in the brain is a complex process that allows the spatiotemporal regulation of the expression of genes involved in many developmental and physiological processes such as neural cell proliferation and differentiation, neuronal migration, and cortical lamination [3,6,7,8,9,10,11,12].

The complex and heterogeneous modulation of gene expression patterns by TH in the central nervous system (CNS) seems to be associated with the regulation of the intracellular availability of TH for neural cells. Experimental studies in rodents have established that TH availability and action in the CNS are tightly controlled by two main factors: TH metabolism by local deiodination, which activates and inactivates TH inside the brain [13,14], and T3/T4 transport across the cell membranes [15].

Although THs are small molecules, in physiological conditions, they cannot cross membranes by passive diffusion due to their amphipathic nature. A body of evidence published from the 1970s onward has demonstrated that TH transfer across the plasma membrane requires a carrier-mediated mechanism and the role of passive diffusion, if any, is limited [16,17]. These studies indicate that the transport of TH into cells is a saturable process that may be inhibited by aromatic and/or aliphatic amino acids. The identity of TH transmembrane transporter proteins started to be elucidated in the late 1990s. Several TH transporters have been described since then, and amongst them, two are widely accepted as being the most important transporters with physiological functions in the CNS: the monocarboxylate transporter 8 (MCT8) [18] and the organic anion transporting polypeptide 1C1 (OATP1C1) [19]. These transporters are included in the MCT and OATP families, respectively, and both belong to the major facilitator superfamily (MFS), whose architecture is generally organized in two symmetrical bundles of six transmembrane domains linked through a large intracellular loop. MFS proteins likely transport substrates according to the “rocker switch model” in which the presence of substrate induces a conformational change that leads to the uptake or efflux of the substrate molecule [20]. MCT8 was cloned in 1994 [21] and it is the only TH transporter specific for T3 and T4 [18]. OATP1C1 mediates with high affinity the transport of T4 and the iodothyronine product from its deiodination, rT3. Indeed, it has the highest affinity for T4 among all the TH transporters [15]. In addition, OATP1C1 transports other substances such as metabolites of steroid hormones, including 17-(β-D-glucuronic acid) estradiol, but virtually no T3 [19].

In a parallel group of discoveries, the identification of human diseases associated with mutations in TH transporters was very relevant and conclusive. For instance, mutations in MCT8 were associated with severe intellectual disability accompanied by a specific TH blood pattern, causing Allan–Herndon–Dudley syndrome (AHDS) or MCT8 deficiency [22,23]. These reports support the physiological relevance of TH transporters and open a new research pathway for TH transport physiology and management. Recently, the first human case of OATP1C1 deficiency was reported, also in the context of progressive neurodegeneration and cerebral hypometabolism [24].

The study of the cellular location of these transporters in brain tissue provides information about the physiology and flow of TH in the neuronal network and the pathophysiology of the neurological symptoms that occur when their functionality is deficient. A summary of previous studies in the brains of several species and ages is given in Table S1. For instance, it is widely accepted that MCT8 is expressed in blood–brain barriers in many species at different ages [25]. Thus, MCT8 is strongly expressed in endothelial cells in all parts of the brain, ependymal cells of the choroid plexus and tanycytes of the third and fourth ventricle in fetal, juvenile and adult mice and rats, neonatal and adult cynomolgus monkeys, and fetal and adult humans [26,27,28,29,30,31,32,33,34,35,36,37,38,39,40], while OATP1C1 is only very weakly expressed or not detected [19,27,29,30,32,34,40,41]. The expression of both transporters in different quantities has been reported in human fetal brain neurons in the cerebral cortex, intermediate zone, subventricular and ventricular zones, hippocampal formation, and brainstem [32,33,34,36,37,40,42], as well as in astrocytes [32,40]. Most of the previous studies coincide in the reporting of higher expression of MCT8 and OATP1C1 in neurons in the fetal and juvenile mouse brain. There are very few studies that analyze the distribution of these transporters in human and monkey neurons, especially at adult stages, and they either report negative results [26] or are very unspecific in terms of defining the specific filiation of the transporter-expressing neuron.

Different types of neurons can be identified in all parts of the brain, and they are very specifically organized in particular in the cerebral cortex. Cortical cytoarchitectonics separates six layers with different cells contents and proportions. Layers II, III, V, and VI have many pyramidal cells, the long- and short-axon-projection cells that connect with other cortical areas, the basal ganglia, thalamus, brain stem and spinal cord, constituting the main pathway for regulation and control of voluntary movements. Additionally, a large number of different types of short-axon inhibitory interneurons are located amongst the pyramidal cells and regulate their activity, modulating their excitation and output through synapses whose locations depend on the kind of interneuron. Among them, *chandelier* cells exert control at the axonal cone of the pyramidal neurons, bipolar cells at the apical dendrites, basket cells at the soma and others such as *bitufted* or *double bouquet* cells at the basal dendrites [43,44]. All these scenarios are constantly regulated by internal trophic inputs generated by factors such as blood/oxygen flow, astrocytic activity, neuropeptides, hormones such as TH and external inputs such as myelinated and unmyelinated thalamo-cortical afferents that are distributed in specific cortical layers (layers III and V in the motor cortex) and activate interneurons and other elements such as pyramidal apical dendrites, respectively. In general, this microcircuitry acts like a “dimmer” for pyramidal neuron activity.

The main aim of this study is to further explore the distribution of MCT8 and OATP1C1 in the neural cellular types; neurons, interneurons and their processes; and glia and their processes, as well as the blood–brain barrier in the motor cortex of humans and monkeys. This will allow us to better understand the role of TH transporters in the functionality of the excitatory–inhibitory dynamics of the motor cortex, from which the final control over movements is exerted. Additionally, knowing the exact nature of cortical cells and elements that express those transporters will enable us to model the etiopathogenesis of transporter-deficient syndromes, since these are known to be associated with a dramatic motor disruption phenotype, often reflected in abnormal cortical excitation inducing epilepsy, hypotonia, spastic tetraplegia and many other movement alterations. Here, we performed immunohistochemical and double labeling immunofluorescence experiments in human and monkey motor cortices for MCT8 or OATP1C1 and several markers to identify the motor cortex microcircuitry cell subpopulations expressing these transporters. We added the study of monkey tissue because there were very few previous data on it, and because we needed to know whether the many differences in terms of cell expression of TH transporters between humans and mice or rats were still present between monkeys and humans. Our findings suggest that a lack of function of these transporters has a significant impact on the cortex’s excitation–inhibition balance.

## 2. Results

We analyzed cellular expression of the TH transporters MCT8 and OATP1C1 in the motor cortices of several adult individuals and monkeys, both males and females, as detailed in Table 1 and Table 2. One specimen of squirrel monkey brain tissue was histologically analyzed. As we only analyzed one monkey from this genus and the results obtained were the same in terms of types of neurons and cells expressing MCT8 and OATP1C1 in all monkeys independently of genus, only data from macaques are shown in the figures and throughout the text. All the figures in this section and in the Supplementary Figures section show the most representative results.

### 2.1. General Distribution of MCT8 and OATP1C1 in the Human and Macaque Adult Motor Cortex

**Figure 1:**
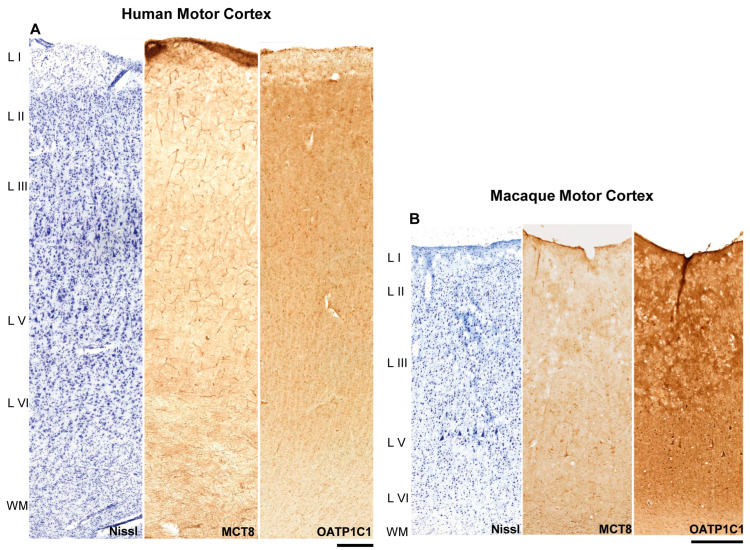
MCT8 and OATP1C1 expression profiles in the motor cortex of humans and macaques. Compositions show representative brightfield photomicrographs taken from adjacent sections of the human (**A**) and macaque (**B**) cerebral motor cortex through layers I to the superficial part of white matter, after Nissl staining (left), immunostaining for MCT8 (middle) and OATP1C1 (right). Note that at this low magnification, large neurons can be observed in layer V of the macaque. MCT8 in humans (**A**) can be observed mainly in blood vessels, while OATP1C1 is observed mainly in cells. In macaque (**B**), however, cells and large vessels express OATP1C1, but not smaller capillaries. L I–VI: layers of the cerebral cortex, WM: white matter. Scale bar = 500 μm (**A**) and 400 μm (**B**).

The general distribution of the expression of MCT8 and OATP1C1 in the human (Figure 1A) and macaque (Figure 1B) motor cortex, through layers I to VI and subjacent white matter, was analyzed in all the individuals and monkeys, and representative results are shown in Figure 1. Cytoarchitectural determination was made following the criteria of Brodmann, 1914 and Vogt and Vogt, 1919 [45,46]. At this low magnification, positive immunostaining signals for both markers could be observed in capillary vessels, cells and fibers, but there were some differences in their intensity within the same tissue section. In the human motor cortex, MCT8 was observed mainly in blood vessels, while OATP1C1 was observed mainly in cells. In the macaque, however, cells and some of the largest vessels expressed OATP1C1, but not smaller capillaries.

In the human motor cortex (Figure 1A), MCT8 cellular expression was observed in each layer. When comparing among layers in the same tissue section, the most intense signal was observed on the surface of layer I. The signal was quite homogeneous in layers II–V and most intense in layer VI. MCT8 was abundantly expressed in the capillaries in all cortical layers.

In the macaque motor cortex (Figure 1B), MCT8 expression was found, as in humans, in every layer of the motor cortex. When comparing among layers of the same cortical tissue section, MCT8 expression was more intense in the surface of layer I. Additionally, a rather homogeneous MCT8 cellular expression was observed in layers II to VI. Giant pyramidal neurons (Betz cells) that were immunopositive for MCT8 were observed at low magnification. In addition, MCT8 was abundantly expressed in the capillaries of all cortical layers.

OATP1C1 expression was found in all layers of the human and macaque motor cortex (Figure 1A,B). When comparing among layers of the same tissue section, the most intense expression was observed in the surface of layer I. Expression in layers II-VI was homogeneous. In general, the gray matter of the cortex exhibited a more intense immunopositive signal than the subcortical white matter found in the same tissue section. Regarding the maximum signal found in each tissue section, the immunopositive signal for OATP1C1 was always more intense for cells than that of MCT8. Small capillaries did not express the protein.

### 2.2. MCT8 and OATP1C1 Are Expressed in the Vessels of the Human and Macaque Adult Motor Cortex

**Figure 2:**
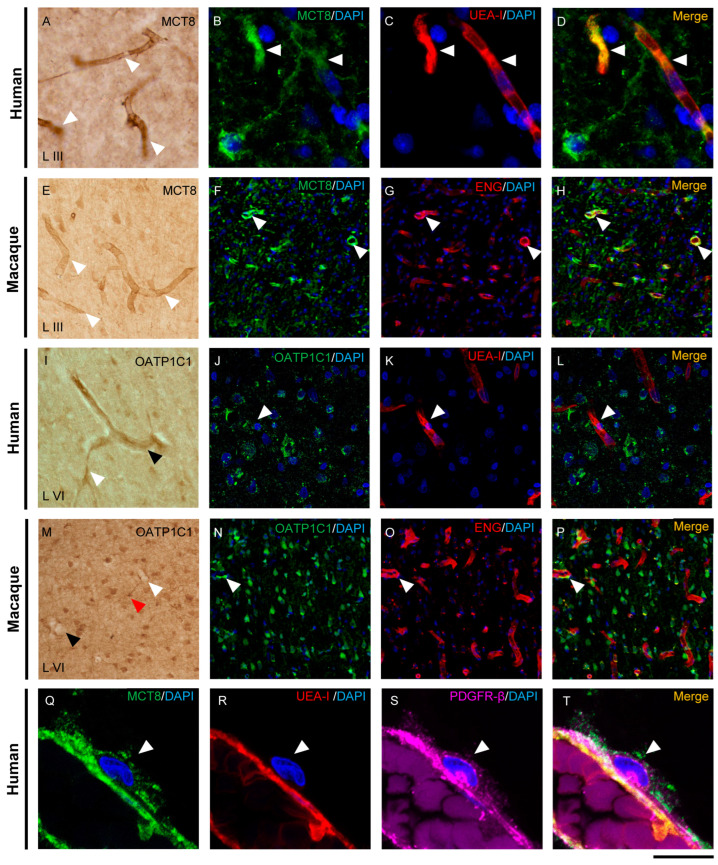
Expression of MCT8 and OATP1C1 in the cerebral barriers of the human and macaque motor cortex. Representative brightfield (**A**,**E**,**I**,**M**) and fluorescence confocal microscope (**B**–**D**,**F**–**H**,**J**–**L**,**N**–**T**) photomicrographs showing immunostaining for MCT8 (**A**–**H**,**Q**–**T**) and OATP1C1 (**I**–**P**) in blood vessels of human and macaque motor cortex. (**A**,**E**) show strong MCT8 staining in the endothelial layer of small vessels and capillaries (white arrowheads) in humans and macaques, respectively. (**B**–**D**,**F**–**H**) show colocalization of MCT8 (green) with the endothelial markers UEA-I (red) and ENG (red). (**I**,**M**) show weak OATP1C1 staining in small vessels (white arrowheads) and venules (black arrowheads). The red arrowhead in M points to a glial cell positive for OATP1C1 with processes surrounding the capillary. (**J**–**L**,**N**–**P**) show colocalization of OATP1C1 (green) with UEA-I (red) and endoglin (red) in a few blood vessels of the human and macaque motor cortex, respectively. White arrowheads point to vessels. In both human and macaque brain tissues, MCT8-immunopositive capillaries are much more abundant than OATP1C1-immunopositive capillaries, which can only be seen occasionally. Moreover, (**Q**–**T**) show MCT8-expressing pericytes (white arrowheads) in the human motor cortex that in addition to express MCT8 (green), also show the pericyte biomarker PDGFR-β (pink), but not the endothelial marker UEA-I (red). Counterstaining with DAPI (blue) shows nuclei of all cells. ENG: endoglin, UEA-I: Ulex Europaeus Agglutinin-I. PDGFR-β: platelet-derived growth factor receptor beta. Scale bar = 100 μm (**A**,**E**,**I**,**M**), 25 μm (**B**–**D**), 50 μm (**J**–**L**), 120 μm (**F**–**H**,**N**–**P**), and 12 μm (**Q**–**T**).

The distribution of MCT8 and OATP1C1 in blood vessels of the human and macaque motor cortex is shown in Figure 2. MCT8 immunopositive signals in capillaries and blood vessels were observed widely distributed in vessels of all diameters in all layers of the human and macaque motor cortex (Figure 2A,E). However, OATP1C1 was more frequently observed in larger vessels and only occasionally observed in small capillaries (Figure 2I,M).

Double-labeling experiments on these transporters with two specific markers for blood vessel endothelial cells, Ulex Europaeus Agglutinin-I (UEA-I) and endoglin, confirmed the localization of MCT8 and OATP1C1 in human and macaque endothelium (Figure 2B–D,F–H,J–L,N–P). We observed that in the human and macaque motor cortex, MCT8 colocalized with the endothelial lectin UEA-I or with endoglin in capillaries and blood vessels, while only a few UEA-I or endoglin immunopositive endothelial cells expressed OATP1C1. In addition, MCT8, but no OATP1C1, was observed in the plasmatic membrane of pericytes around blood capillaries colocalizing with the pericyte marker platelet-derived growth factor receptor beta (PDGFR-β) [47] (Figure 2Q–T). In summary, in the macaque and human motor cortex, we demonstrate that MCT8 is expressed in the endothelial cells of capillaries and blood vessels of different sizes and in the pericytes, whereas OATP1C1 is expressed in some endothelial cells of larger size blood vessels.

### 2.3. MCT8 and OATP1C1 Are Expressed in Pyramidal Neurons of the Human and Macaque Adult Motor Cortex

**Figure 3:**
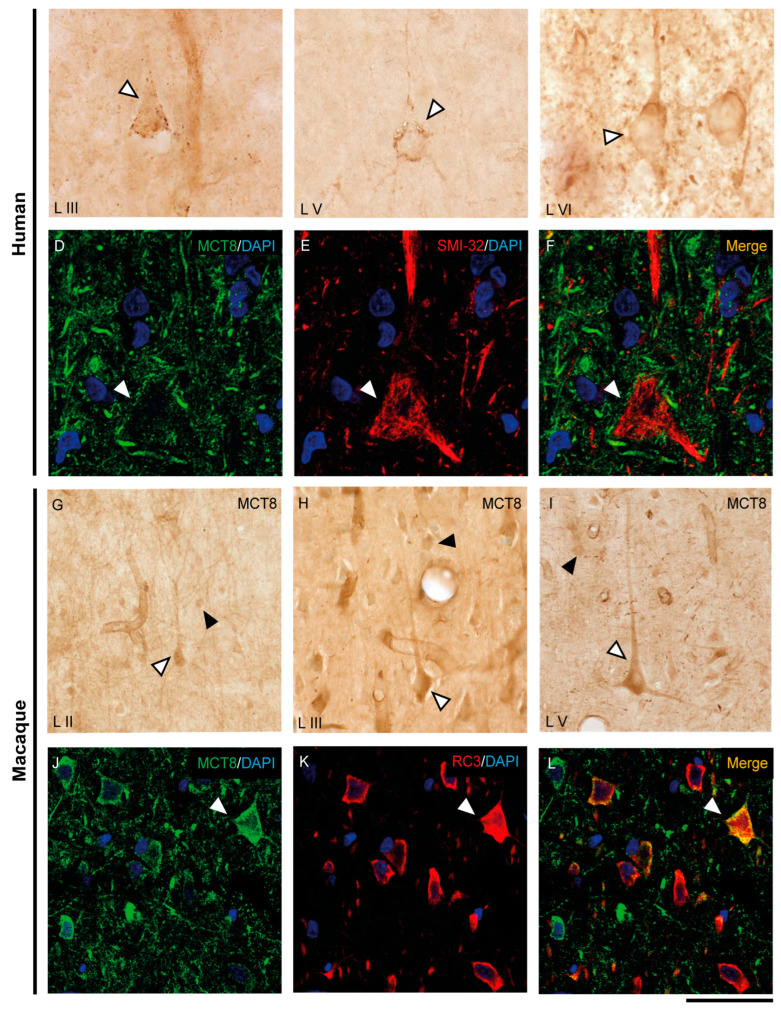
Expression of MCT8 in pyramidal neurons of the human and macaque motor cortex. Representative brightfield photomicrographs show immunostaining for MCT8 in layers III (**A**), V (**B**) and VI (**C**) of the human motor cortex, and layers II (**G**), III (**H**), and V (**I**) of the macaque motor cortex. White arrowheads point to pyramidal neurons with immunopositive signal in the soma, apical and basal dendrites. Black arrowheads point to smaller cells with the morphology of interneurons. (**D**–**F**) (human) and (**J**–**L**) (macaque) show confocal microscope images from double-stained sections for MCT8 (green) and the pyramidal neuron markers SMI-32 (red) for 200 kDa neurofilament protein or neurogranin/RC3 (red), respectively. White arrowheads point to pyramidal neurons. Counterstaining with DAPI (blue) shows nuclei of all cells. Note that in humans, the MCT8 signal is located mainly in the pyramidal cell membrane, while in macaques it is located in the membrane and the cytoplasm. Scale bar = 50 μm (**A**), 120 μm (**B**,**I**), 25 μm (**C**–**F**), 60 μm (**G**), 62.5 μm (**H**), and 43.5 μm (**J**–**L**).

**Figure 4:**
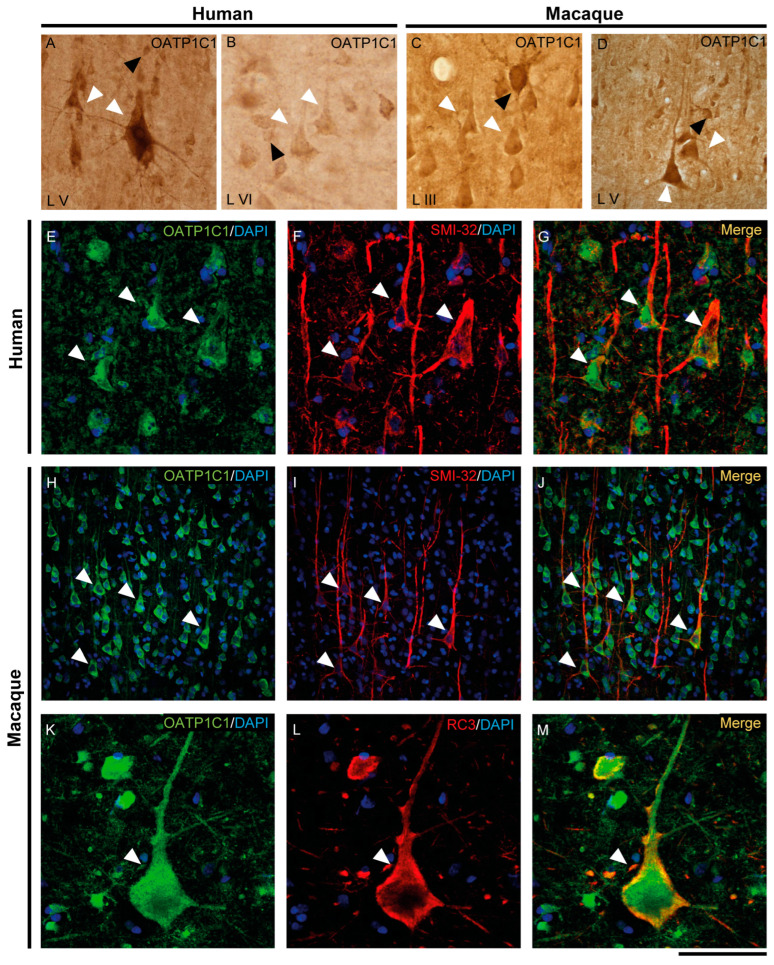
Expression of OATP1C1 in pyramidal neurons of the human and macaque motor cortex. Representative brightfield photomicrographs show immunostaining for OATP1C1 in layers V (**A**) and VI (**B**) of the human motor cortex and layers III (**C**) and V (**D**) of the macaque motor cortex. (**E**–**G**) Confocal microscope images for OATP1C1 (green, **E**) and the pyramidal neuron marker SMI-32 (red, **F**) immunostaining in the human cortex. (**G**) Merged image showing the colocalization of both signals. (**H**–**M**) Confocal microscope images for OATP1C1 (green, **H**,**K**), SMI-32 (red, **I**) and neurogranin/RC3 (red, **L**) immunostaining in the macaque cortex. (**J**,**M**) Merged confocal photos, respectively, showing the colocalization of the signals. Counterstaining with DAPI (blue) shows nuclei of all cells. White arrowheads point to pyramidal neurons. Note that the signal can be identified in the membrane, cytoplasm, and apical and basal dendrites. Black arrowheads point to neurons with interneuronal morphology. L III–VI: layers of the cerebral cortex. Scale bar = 120 μm (**A**,**D**,**H**–**J**), 60 μm (**B**,**C**), 40 μm (**E**–**G**), 50 μm (**K**–**M**).

The expression of MCT8 and OATP1C1 in pyramidal neurons of the human and macaque motor cortex is shown in Figure 3 and Figure 4. In the human motor cortex, weak MCT8 immunostaining signal is present in the cell membrane, apical and basal dendrites of pyramidal neurons of different sizes in layers II-VI (Figure 3A–C). In the macaque motor cortex, MCT8 immunostaining signal is seen in small to large pyramidal neurons in layers II-VI (Figure 3G–I), with a homogeneous signal in the apical and basal dendrites and inside the cytoplasm. In particular, MCT8 is present in giant pyramidal neurons (Betz cell) in layer V in humans (Figure 3B) and macaques (Figure 3I). On the other hand, OATP1C1 immunopositive signal is very high in pyramidal cells of various sizes in layers II-VI of human and macaque motor cortex, which are intensely marked in the membrane, cytoplasm, and in their apical and basal dendrites (Figure 4A–D). The signal in giant pyramidal cells of layer V is particularly evident and all processes are immunopositive (Figure 4A,D).

We have shown that the TH transporters MCT8 and OATP1C1 in human and macaque are expressed in neurons with a clear pyramidal morphology. However, we also identified them via pyramidal neuron biomarkers. Figure 3 and Figure 4 show the coexpression of these transporters with the 200 kD neurofilament labeled by SMI-32 [48] (Figure 3D–F for MCT8 and Figure 4E–J for OATP1C1) and with neurogranin/RC3 [49] (Figure 3J–L for MCT8 and Figure 4K–M for OATP1C1) via double-labeling immunofluorescence and posterior identification with confocal microscopy. Colocalization of MCT8 and SMI-32 is well observed in macaques, but in humans, the cellular MCT8 signal is restricted to the membrane, as expected from the results previously seen with DAB staining. The immunostaining signal of OATP1C1 (Figure 4) is very intense in cortical cells in human and macaque, and, therefore, its colocalization with SMI-32 and RC3 in the soma and dendrites of pyramidal neurons as well as basal dendrites is very clear.

### 2.4. MCT8 and OATP1C1 Are Expressed in GABAergic Interneurons of the Human and Macaque Adult Motor Cortex

**Figure 5:**
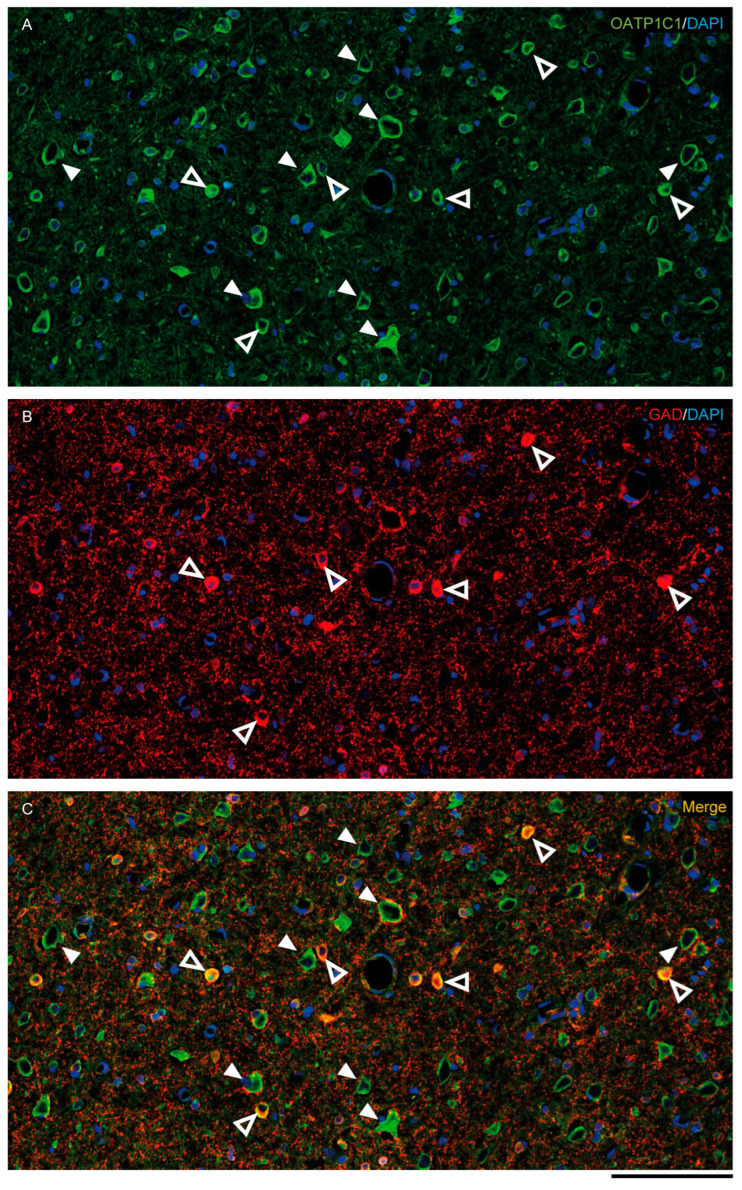
Expression of OATP1C1 in the macaque cortical GABAergic neurons. Confocal tile scan microscope images of a macaque cortical section double-stained for OATP1C1 (green, **A**) and the interneuronal marker GAD (red, **B**). (**C**) Merged image showing the colocalization of both markers only in small cells surrounding pyramidal neurons. Counterstaining with DAPI (blue) shows nuclei of all cells. White arrowheads point to pyramidal neurons. Hollow white arrowheads point to double-labeled interneurons. Note that OATP1C1 is expressed in practically all GAD-immunopositive neurons. GAD: glutamic acid decarboxylase. Scale bar = 100 μm.

**Figure 6:**
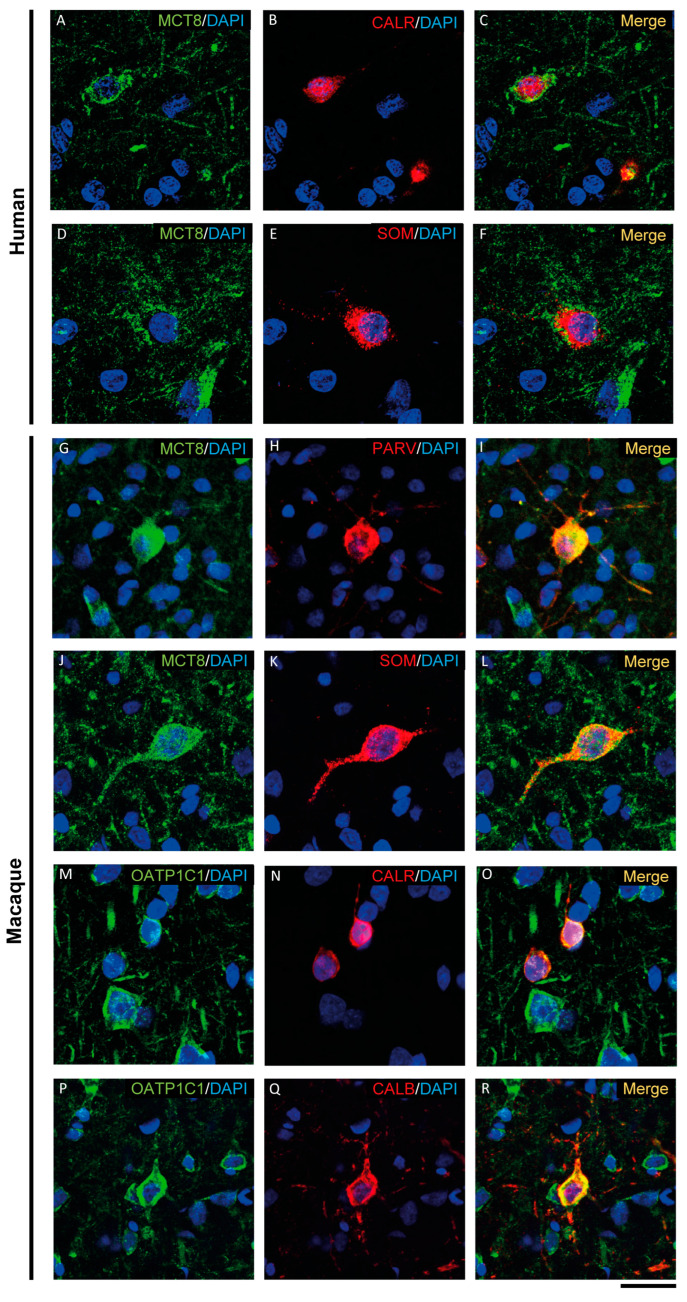
Expression of MCT8 and OATP1C1 in different subpopulations of GABAergic interneurons. Representative confocal microscope images of human (**A**–**F**) and macaque (**G**–**R**) cortical sections double-stained for MCT8 or OATP1C1 and several interneuronal markers. (**A**–**C**) Coexpression of human MCT8 (green) with CALR (red). (**D**–**F**) Coexpression of human MCT8 (green) with SOM (red). (**G**–**L**) Colocalization of macaque MCT8 (green) with PARV (red, **G**–**I**) and SOM (red, **J**–**L**). (**M**–**R**) Colocalization of macaque OATP1C1 (green) with CALR (red, **M**–**O**) and CALB (**P**–**R**). Counterstaining with DAPI (blue) shows nuclei of all cells. CALB: Calbindin-D-28K, CALR: calretinin, PARV: parvalbumin, SOM: somatostatin. Scale bar = 50 μm.

MCT8 and OATP1C1 are expressed in GABAergic interneurons in the adult human and macaque motor cortex, as shown by their colocalization in cells immunopositive for glutamic acid decarboxylase (GAD), the synthesizing enzyme for the GABA neurotransmitter (Figure 5 and Figure S1). While the majority of GAD interneurons express OATP1C1 (Figure 5A–C and Figure S1C1–C3), just some of them express MCT8 (Figure S1A1–B3). MCT8 and OATP1C1 colocalize with the interneuronal subtype markers calretinin (CALR) [50,51] (Figure 6A–C,M–O), somatostatin (SOM) [51] (Figure 6D–F,J–L), parvalbumin (PARV) [50,51,52] (Figure 6G–I), and neuronal nitric oxide synthase (nNOS) [53] (Figure S1D1–G3) in humans and macaques, but colocalization with calbindin (CALB) [50,51,52] only happens in monkeys (Figure 6P–R).

Thus, according to the neuronal morphological characteristics and the colocalizing markers specific for GABAergic interneuron subpopulations [44,50,51,54], we can specify that MCT8 is expressed in basket, *bitufted* and bipolar cells in the human motor cortex, while in the macaque motor cortex, MCT8 is expressed in basket, *bitufted*, multipolar and bipolar cells. On the other hand, OATP1C1 is observed to be expressed in basket, bipolar and *Martinotti* cells in the human motor cortex, while it is expressed in basket, *bitufted* and bipolar cells in the macaque motor cortex.

### 2.5. MCT8 and OATP1C1 Are Expressed in Glia and Fibers

**Figure 7:**
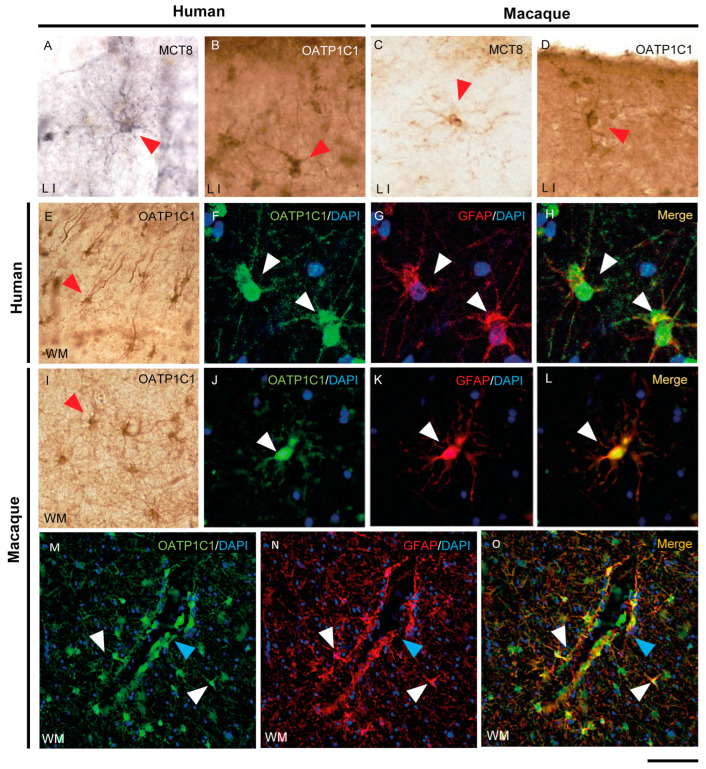
Expression of MCT8 and OATP1C1 in human and macaque motor cortex astrocytes. Representative brightfield (**A**–**E**,**I**) photomicrographs of sections immunostained for MCT8 (**A,C**) and OATP1C1 (**B,D,E,I**) in cortical layer I (**A**–**D**) and subjacent white matter (**E**,**I**). Confocal microscope images of human (**F**–**H**) and macaque (**J**–**O**) sections double-labeled for OATP1C1 (green) and GFAP (red). Counterstaining with DAPI (blue) shows nuclei of all cells. Note that there is full colocalization of both markers. White and red arrowheads point to astrocytes. Blue arrowheads point to a blood vessel. GFAP: glial fibrillary acidic protein. Scale bar = 50 μm (**A**–**D**,**J**–**L**), 110 μm (**E**,**I**), 25 μm (**F**–**H**), and 78 μm (**M**–**O**).

The expression of MCT8 and OATP1C1 in glial cells in the human (Figure 7A,B,E) and macaque (Figure 7C,D,I) motor cortex is easily identified via morphological characteristics through immunohistochemistry. The type of glial cells expressing MCT8 and OATP1C1 in human and macaque motor cortices was analyzed in double-labeling immunofluorescence experiments with glial fibrillary acidic protein (GFAP) [55] to define astrocytes (Figure 7F–H,J–O and Figure S2A1–B3), myelin basic protein (MBP) to define oligodendrocytes [56] (Figure S2C1–F3), and ionized calcium binding adaptor molecule 1 (IBA1) to define microglia [57] (Figure S2G1–J3). The immunopositive signal of OATP1C1 clearly colocalizes with the GFAP signal of the soma and the processes of human and macaque motor cortex astrocytes in grey and white matter, as did MCT8 in the macaque. We also observed many images of astrocytes immunopositive for OATP1C1 surrounding microvessels (Figure 7M–O). While OATP1C1 was observed in some populations of microglia in the human and macaque motor cortex (Figure S2I1–J3), MCT8 was not present in these cells (Figure S2G1–H3). Most MCT8 expression did not colocalize with MBP in the somas of oligodendrocytes in human and macaque motor cortices, neither in gray nor white matter, but some of the MCT8 immunopositive fibers were surrounded by MBP signal, indicating that MCT8 is expressed in myelinated fibers (Figure S2C1–D3). Concerning OATP1C1, it colocalized weakly with MBP in oligodendrocytes, and it could be observed in fibers surrounded by MBP immunoreactivity in human and macaque motor cortices, indicating that OATP1C1 is expressed in myelinated fibers and oligodendrocytes (Figure S2E1–F3).

In summary, among the major glial cell populations of the human and macaque motor cortex, MCT8 is mainly found in astrocytes and myelinated fibers, while OATP1C1 is relatively widespread and found in astrocytes, oligodendrocytes, microglia and myelinated fibers.

### 2.6. OATP1C1 Is Expressed in Corpora Amylacea

**Figure 8:**
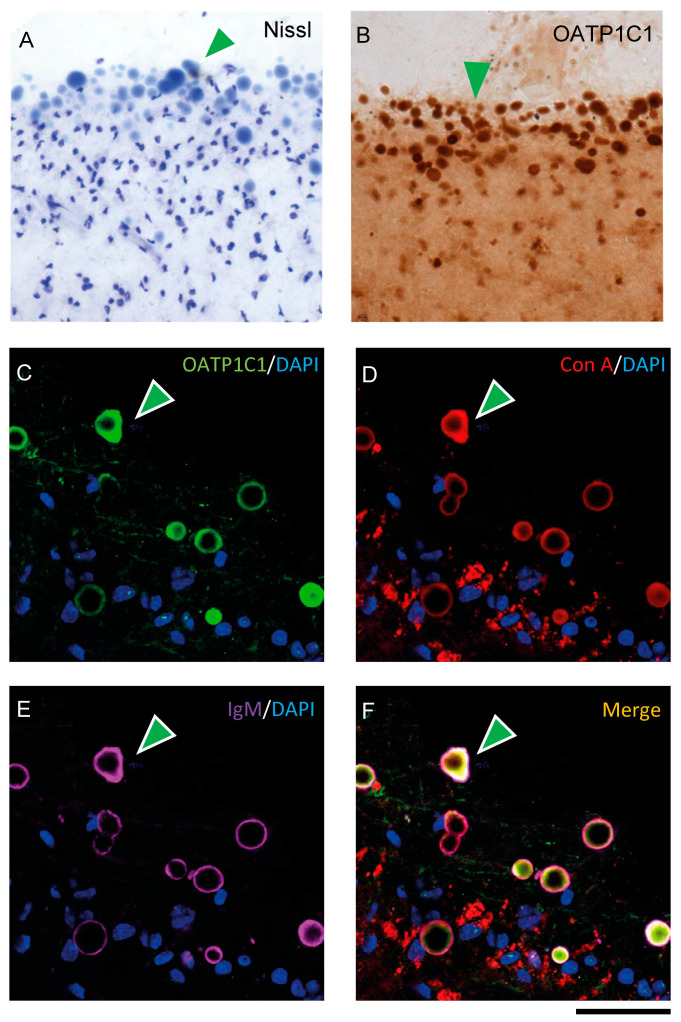
Expression of OATP1C1 in *Corpora amylacea* of the human motor cortex. (**A**,**B**) Representative brightfield photomicrographs of Nissl-stained (**A**) and OATP1C1 immunostained (**B**) sections in which green arrowheads point to numerous spherical vesicles located in the subpial region and layer I of the human motor cortex. (**C**–**F**) Confocal microscope images of triple labeling for OATP1C1 (green, **C**), and the two *Corpora amylacea* markers Con A (red, **D**) and IgM (purple, **E**). The merged image (**F**) shows the total colocalization of the three markers (green arrowheads). Counterstaining with DAPI (blue) shows nuclei of all cells. Note that the vesicles do not contain a blue nucleus. Con A: Concanavalin A. Scale bar = 50 μm (**A**,**B**), 40 μm (**C**–**F**).

When performing immunohistochemistry for OATP1C1, we unexpectedly observed that in all seven human individuals, the motor cortex surface presented a large number of aggregated spherical vesicles with strong OATP1C1 immunoreactivity (Figure 8B and Figure S3A–D). These vesicles varied greatly in size and tended to be located in the most superficial zone of layer I, but could also be seen in deeper layers. These vesicles were also Nissl-stained (Figure 8A) and were not observed in any macaque motor cortex analyzed. The OATP1C1 expression in these vesicles was observed, without antigen retrieval, using four different anti-OATP1C1 antibodies against different epitopes of OATP1C1 protein, including the antibody donated by Dr. Visser [58] (Figure S3A–D). Only the single mouse-derived sc-398883 Santa Cruz antibody (which did not work without antigen retrieval) and the PA5-115919 antibody from Invitrogen (which never worked at all in our conditions) failed to label these vesicles.

We tested the possibility that these vesicles could be *Corpora amylacea*, vesicular outgrowths produced in astrocytes that could function by entrapping waste products and can be released from the brain to the cerebrospinal fluid (CSF) and the lymphatic system [59,60]. To test this, we performed double- and triple-labeling experiments for OATP1C1 and several markers specific for *Corpora amylacea*: Immunoglobulin M (IgM) antibodies that recognize the neo-epitopes of IgM [61] and concanavalin A (Con A), which recognize hexoses including mannose and glucose [62]. OATP1C1 coexists with Con A and IgM in *Corpora amylacea*. IgM is usually found outside *Corpora amylacea* and Con A and OATP1C1 are habitually found inside, as confirmed with 3D reconstruction (Figure 8C–F and Video S1). The experiments were performed using specific secondary antibodies against the C region of the γ1 chain of rabbit IgG to label rabbit anti-OATP1C1 antibodies in order to exclude the possibility of false positives in *Corpora amylacea* immunostaining due to the contamination of IgM in commercial antibodies.

To further characterize the nature of these vesicles, we analyzed the expression of GFAP as a marker of *Corpora amylacea* astrocytic origin [60], as well as the expression of several neurofilaments that have been previously defined as components of these vesicles [63,64] such as 68kDa neurofilament protein and 200 kDa neurofilament protein using specific antibodies (NF68 and SMI-32, respectively). We also analyzed the presence of phospho-Tau with the specific antibody AT8. All of these proteins were present in these vesicles, as is shown in adjacent sections (Figure S3E–I). Importantly, these vesicles were negative for the β-amyloid specific Congo Red staining (Figure S3M,N), which is consistent with the earlier findings [65,66]. Using the same experimental conditions as for OATP1C1 immunohistochemistry, *Corpora amylacea* were not positive for UEA-I (Figure S3K–L) and did not express MCT8, CALB, PARV, CALR, ChAT, SOM, IBA1, MBP or nNOS.

In summary, we used several experiments to identify that the intensely positive OATP1C1 spheric vesicles that we found in layer I of the human motor cortex are *Corpora amylacea*, which express GFAP of astrocyte origin, hexoses including mannose and glucose, neurofilaments NF68, NF160 and SMI-32 and scarce phospho-tau but do not contain amyloid and L-fucose.

## 3. Discussion

In this work, we studied the nature of the cellular elements in which the TH transporters MCT8 and OATP1C1 are expressed in the motor cortex of healthy adult primates, including humans and macaques. The aim of this study is to understand the role of these transporters in the excitation–inhibition balance of motor cortex microcircuits, and, thus, to improve the understanding of the consequences of their absence in these circuits, to better understand the pathophysiology of MCT8 and OATP1C1 deficiency and to provide a base of knowledge for future therapeutic applications oriented toward cellular targets.

### 3.1. Study Limitations

The histological analysis of the structure of the primate and human nervous system presents several limitations ”per se”. Apart from the ethical issues that need to be fully complied with, studies on human postmortem material by means of biological techniques are usually influenced by certain factors such as donor status, condition of tissue source, cryoprotection, storing and processing. All of those might change the way antigens are exposed to specific antibodies used to detect TH transporters. Regarding the first factor, donor status, we selected those brain tissues whose donor ages were either adulthood (29, 32, 55 and 59 years old) or aged (86, 97 and 98 years old) considered normal upon macroscopical and microscopical examination by a pathologist and a neurologist. Some of them, not all of them, died after conditions that could be associated with non-thyroidal illness syndrome. However, it is known that in that syndrome, even when T3 might be reduced, human expression of MCT8 does not vary in liver and muscle, nor does there exist any study on the expression of MCT8 and OATP1C1 in the human cerebral cortex in those conditions [67]. In murine animal models, expression of MCT10 and OATP1C1 is reduced in the hypothalamus, but not MCT8 [68]. In any case, even if the expression of these transporters would be altered by non-thyroidal illness syndrome in our human tissue, they were still present and both presented a similar immunocytochemical signal in the subpopulations of cells described in the paper, as we have demonstrated consistently, in all of the studied brains.

Regarding other factors, we can affirm with certainty that all human and macaque tissue extraction and storing was exactly the same for all individuals, and that all human tissue was collected, cryoprotected and stored using a common protocol. Fixation of human tissue takes a long time, and its effects are distinct in each specimen depending on pre- and postmortem conditions. This can lead to variability in the final production of the immunocytochemical signal that cannot be completely controlled. Concerning the processing factor, we invested a lot of effort in optimizing all possible agents to eliminate non-specific staining, autofluorescence and false negatives to yield reliable results. We used the same lot of the polyclonal anti-MCT8 antibody that had been validated in terms of specificity through previous studies in post-mortem analysis of an MCT8-deficient fetus [40] and three other lots from the same batch that produced similar results. In addition, we used another commercially available MCT8 antibody that had also been reported previously (NBP2-57308 Novus^®^) [26], but we discarded it after confirming that it produced an intracellular nonspecific signal. For the immunocytochemical detection of the OATP1C1 transporter, we tested the OATP1C1 antibodies kindly given by Dr. Visser [58], and we purchased five other OATP1C1 commercial antibodies on the market (Table S2). All of them were specific for human epitopes except for the immunogen of the sc-398883 Santa Cruz^®^ antibody, which was mouse-derived and worked properly after antigen retrieval techniques. One of the three batches of the Cusabio^®^ OATP1C1 antibody produced non-specific staining in many nuclei, so these results were not accepted. PA5-115919 Invitrogen^®^ did not work at all. In summary, the results shown in this study are based on Abcam^®^, two batches of Cusabio^®^ and Santa Cruz^®^ and Visser antibodies, which produced specific signals.

Further verification of the expression distribution of OATP1C1 using other methods, such as in situ hybridization, should be performed, although the mRNA integrity and preservation in post-mortem human tissue are not always granted and errors in signaling would be very likely.

### 3.2. TH Transporters in the Blood–Brain Barrier

We investigated the expression of MCT8 and OATP1C1 in vessels in the human and macaque motor cortex. As documented in this study, MCT8 is very abundant mainly in the endothelial cells of small capillaries and venules, while OATP1C1 is only rarely found in the endothelium of larger vessels. This is fully coincident with previous results by Zhang et al. (2016) obtained by means of RNA sequencing of brain-purified endothelial cells [32] and also coincident with immunofluorescent results observed by Roberts et al. (2008) and Wilpert et al. (2020) in the human brain [26,29], with immunohistochemical results observed by Wirth et al. (2009) in human brain [36], Chan et al. (2014) in the human fetal and adult brain [33] and López-Espíndola et al. (2019) in the fetal brain [40], as well as with liquid chromatography tandem mass spectrometry results detected by Ito et al. (2011) in the macaque brain [31]. 

Interestingly, a novel finding described in our study is the presence of MCT8, but not OATP1C1, in pericytes. This result was very consistent and very clear and was documented with colocalization studies with pericyte biomarker, PFGFR-β. Wilpert et al. [26], however, did not detect the expression of MCT8 in pericytes; the discrepancy with our data could be based on differences in tissue sample processing and/or the antibodies used. Pericytes (previously known as Rouget cells) [69] are multi-functional mural cells of microcirculation that wrap around endothelial cells [70]. Pericytes are embedded in the basement membrane of blood capillaries, where they communicate with endothelial cells by means of both direct physical contact and paracrine signaling [71]. Brain pericytes are a key component of the neurovascular unit [72,73], help to maintain homeostatic and hemostatic functions and sustain the blood–brain barrier [74]. Pericytes regulate capillary blood flow [75,76,77,78] and the clearance and phagocytosis of cellular debris in vitro [79]. Pericytes stabilize and command the maturation of endothelial cells and their junctions [80]. A deficiency of pericytes in the CNS can cause increased permeability of the blood–brain barrier [74]. Recent reports support the regulation by TH of the cell proliferation marker CDC-47 and the vascular endothelial growth factor (VEGF) in pericytes and endothelial cells [81], suggesting that TH differentially affects the levels of proliferative activity, angiogenesis and apoptosis in these elements of the blood–brain barrier. Our results support this idea with the novel contribution of the finding of MCT8 transporter in pericytes in both humans and macaques.

### 3.3. TH Transporters in Glial Cells

Our results corroborate the findings of the previous analysis by Zhang et al. using RNA sequencing, where it was found that MCT8 is expressed in purified human astrocytes and to a lesser extent in oligodendrocytes, whereas OATP1C1 is expressed at a high level in astrocytes and microglia [32]. They are also coincident with the immunochemical results observed by Alkemade et al. in adult human brains [37], in situ hybridization results observed by Wittman et al. in rat and mouse brains [27], and the immunochemical and immunofluorescent results observed by López-Espíndola et al. in the fetal brain [40]. The role of astrocytes in TH transport and deiodination has been extensively described previously [14,40]. On the other hand, microglia can be regulated by THs [82]. To date, the TH transporters OATP4a1, LAT2 and MCT10 have been reported in microglia [83]. We demonstrated for the first time via double immunofluorescence that OATP1C1, but not MCT8, was present in small amounts of microglia in the human and macaque motor cortex. Although the role of TH transporters in modulating the availability of TH for glial cells in conditions of brain injury needs to be further investigated [84], our results suggest that OATP1C1 could be involved in controlling TH bioactivities in microglia, such as microglial migration and phagocytosis [85,86].

### 3.4. TH Transporters in Cortical Neurons

Our study provides the first immunocytochemical demonstration of the expression of MCT8 and OATP1C1 in pyramidal neurons and GABAergic interneurons throughout all layers of the human and macaque motor cortex. Previous studies demonstrated MCT8 and OATP1C1 expression in immature neurons in the developing cerebral cortex [33,36,40]. However, while research on the distribution of these two transporters has increased in recent years, there are still few anatomic reports regarding the expression of MCT8 and OATP1C1 protein in the adult human cortex and even less information for the adult macaque cortex.

A relevant contribution of our paper is the attempt at identifying the cortical neuron subtypes that express MCT8 or OATP1C1 by their biomarkers. Thus, we have demonstrated colocalization of MCT8 and OATP1C1 with RC3/neurogranin, which is a substrate of calmodulin-binding protein kinase C expressed mainly in pyramidal cells in the cerebral cortex [49] and gives rise to many forms of synaptic plasticity. RC3 mRNA is down-regulated under hypothyroid conditions [87], and the protein RC3/neurogranin is a direct target of TH in the human brain [88]. Our results support that the expression of OATP1C1 and/or MCT8 in pyramidal cells is necessary to modulate RC3 expression by TH. It has also been documented here that MCT8/OATP1C1 expressing pyramidal cells also express neurofilaments of different molecular weights, including 200kd, which is a differential feature of long-projection pyramidal neurons. Pyramidal neurons are abundant in the cerebral cortex of virtually all mammals [89], birds, fish and reptiles. So important is the role of these neurons in the motor system that any alteration in them, such as lack of trophism and/or defects in synaptic plasticity caused by TH deficiency due to missing transporter(s), produces the typical symptoms of upper motor neuron disease, which is present in AHDS patients as bilateral Babinski reflexes and hyperreflexia [90] and in the patient with OATP1C1 deficiency as spasticity of the lower limbs [24].

When comparing the qualitative importance of one or the other transporter in the motor cortex, we can state that, overall, OATP1C1 is the major and most abundant TH transporter in pyramidal neurons. We are aware that the quantification of immunosignal is not reliable in terms of stating abundance of the studied transporters, so we performed qualitative comparisons in intensity of immunolabeling between different structures in the same tissue sections. In those terms, MCT8 is present in most pyramidal neurons, although it is less abundant and provides a weaker immunofluorescence signal than the signal detected in the endothelial cells. One of the intriguing but consistent facts observed in our study is the differential cellular location and signal of the MCT8 protein between humans and macaques. Both human and macaque pyramidal cells have their proteins located in basal and apical dendrites, axonal cones and soma. However, human pyramidal cells tend to have it localized in the soma membrane, while those of monkeys contain protein in the membrane and the cytoplasm as well. Moreover, MCT8 immunopositive signal is more abundant in the macaque. This may indicate either that macaque pyramidal cells require more TH than humans or the participation of other additional TH transporters in human neurons. On the other hand, the N-terminal end of the MCT8 protein of 539 amino acids contains one PEST sequence (proline, glutamic acid, serine and threonine) and the N-terminal end of the MCT8 protein of 613 amino acids contains two PESTs [21]. Generally, PEST is considered to be associated with rapid protein degradation [91]. As mentioned above, the differences between human and macaque postmortem tissue processing and MCT8 being a protein sensitive to degradation could impact MCT8 expression results in the human brain [92]. Finally, concerning OATP1C1, we have not found major differences in its cellular expression between humans and macaques.

As we have demonstrated in this study, pyramidal cells are rich in. A possible function of OATP1C1 in pyramidal cells in normal conditions, based on OATP1C1 transport properties of T4 in-and-out across the cell membranes, would be to provide an intracellular T4 storage mechanism so that when T3 is required, T4 can be released into the extracellular space and thus be available to the surrounding astrocytes, to be deiodinated to T3 [14]. This is in line with some recent suggestions about T4 being more than just a preparatory hormone [93,94]. Both serum hypothyroidism and mice after carotid artery ligation maintain a stable TH environment in the brain for a period of time, which suggests that in addition to TH coming from the peripheral circulation, it is likely that TH is stored in the brain′s own environment and used to maintain homeostasis under certain specific circumstances. In addition, the elevated expression of OATP1C1 in the pyramidal cells could be controlling in normal conditions the availability of the extracellular T4, and subsequently, its non-genomic actions by binding to plasma membrane receptors. The main non-genomic actions described for T4 relate to the modulation of different intracellular signaling pathways, as well as Na^+^-dependent transport systems, at the level of the plasma membrane integrin αvβ3 receptors [95,96]. Additionally, in OATP1C1-expressing neurons, the extracellular T4 could be uptaken by OATP1C1 to facilitate T4 deiodination to reverse T3 (rT3) by the action of DIO3, a deiodinase that is mainly expressed in neurons in the adult brain [97], although during CNS development and under pathophysiological conditions it can also be expressed in different cerebral regions and neural cells [98]. Afterward, OATP1C1 could also mediate the cellular efflux of the generated rT3 to the extracellular space.

OATP1C1 being more specific for T4 than for T3, it is difficult, for now, to determine whether the presence of OATP1C1 in pyramidal cells could compensate for the lack of T3 transport in the absence or dysfunction of MCT8 in these cells. In this case, the expression of OATP1C1 in pyramidal cells provides the possibility that T4 may be internally deiodinated to T3, although this is still a hypothesis with the existing data. The expression of DIO2, the enzyme responsible for the conversion of T4 to T3 that generates the local levels of the nuclear active hormone, is mostly expressed in glial cells, astrocytes and tanycytes [14] in rats, and not in neurons, although it has also been found in interneurons in conditions of cerebral hypothyroidism [99]. In humans, DIO2 is abundantly expressed in the human cerebral cortex [100], but only in astrocytes and not in pyramidal cells nor in interneurons [101,102]. Without DIO2 in neurons, OATP1C1 as a T4 transporter would not compensate for the biological functions of TH, since it is hardly present in the blood–brain barriers. That being said, if T4 could enter the brain through the scarce transporters found in larger vessels or through other TH transporters, a plausible alternative relies on the fact that TRα1, the predominant isoform of TH receptors that exhibits a much stronger response to T4 than TRβ1, is distributed in almost all neurons of the brain [103,104]. This mechanism could account for some few of the TH actions complementary to those dependent on MCT8 transport in normal conditions and in the absence of MCT8-transported T3, sufficient enough to allow some development of the neural tissue.

However, in conditions of human MCT8 deficiency, the neurological syndrome caused by a lack of TH throughout all development shows that the malfunctioning of pyramidal cells is not been compensated for by the T4 transporter OATP1C1, MCT8 being crucial for TH transport across brain barriers. On the other hand, the OATP1C1 human deficient syndrome also shows a dramatic motor cortex disturbance. Therefore, the expression of MCT8 in primate pyramidal cells, although low in comparison with OATP1C1, may have a function that cannot be substituted by OATP1C1. This would also help towards understanding why humans lacking a functional OATP1C1 have such severe neurological symptoms.

Another important and original contribution of our study is the demonstration of the coexpression of MCT8 and OATP1C1 transporters in GABAergic interneurons and the definition of the types of interneurons that express them. Prior studies have shown that the development and differentiation of GABAergic interneurons depends on the regulation of TH [105], and in the *Mct8/Oatp1c1* and *Mct8/Dio2* double KO mouse as well as in an avatar MCT8 deficient mouse, the GABAergic interneurons were altered [106,107,108]. We have shown that MCT8 and OATP1C1 are present in interneurons that inhibit pyramidal cell activity either at the axonal cone such as *chandelier* cells, or at the soma and basal dendrites such as basket cells, or at the apical dendrites such as bipolar cells. GABAergic interneuron inhibition at the cortical microcircuitry is important to modulate the efferent output of the motor system, but synaptic inhibition is not the only function of those neurons. Many sorts of interneurons deliver neuropeptides, such as neuropeptide Y, somatostatin, substance P and cholecystokinin [109]. Those neuropeptides also contribute to trophism, oxygen flow, synaptic development and many other actions [109,110], subsequently modulating the general scenario in which pyramidal neurons execute motor commands. In terms of qualitative distribution and comparison of MCT8 and OATP1C1 in the same section, we generally found more expression of OATP1C1 than of MCT8 both in humans and in monkeys. All the considerations that could explain these differences have been mentioned above.

### 3.5. TH Flow in the Motor Cortex

**Figure 9:**
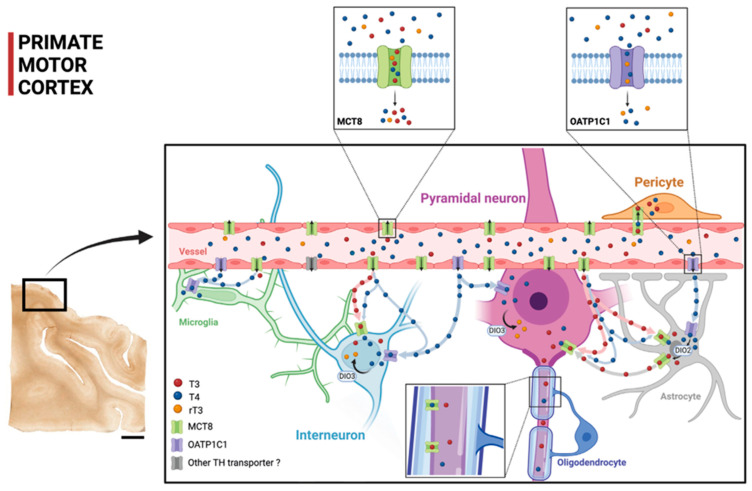
Model of TH transport in human and primate motor cortex based on our contribution. The two small frames above show the TH transport affinities for MCT8 and OATP1C1, with MCT8 facilitating primarily the transport of T3 and T4 and OATP1C1 facilitating the transport of T4 and rT3 (see legend for color key). The scheme shows the distribution of the transporters MCT8 and OATP1C1 and the possible flow of T3 and T4 in the human and non-human primate motor cortical cells. Circulating T3 and T4 pass through the vascular endothelium to the brain parenchyma, relying primarily on MCT8 and on, to a much lesser extent, OATP1C1 and other transporters. Then, T3 and T4 are transported to the intracellular medium by MCT8 and/or OATP1C1 in different target neural cells. T3 enters into astrocytes and some interneurons and pyramidal cells (more abundantly in non-human primates). T4 is converted into T3 by astrocytic DIO2, providing additional T3 to nearby neural cells. DIO3 at the neuronal cell membrane catalyzes the conversion of T4 to rT3 and T3 to T2. T4 enters into neural cells, where it can be stored. DIO2: Deiodinase 2, DIO3: Deiodinase 3. Scale bar = 4000 μm (Created with BioRender.com).

Figure 9 shows a diagram that summarizes our proposal of TH transport within the cortical microcircuitry based on our contribution on the cellular location of MCT8 and OATP1C1 in human and monkey motor cortices, together with previous findings. The figure shows that TH are transported through the blood–brain barrier using MCT8 and OATP1C1 in such a way that MCT8 facilitates the transport of both T3 and T4, whereas OATP1C1 almost only transports T4. The scheme shows the distribution of T3 and T4 transporters MCT8 and OATP1C1 as found in this study and the possible flow of T3 and T4 in motor cortical cells. Circulating T3 and T4 pass through the vascular endothelium to the brain parenchyma, relying primarily on MCT8 and a tiny quantity of OATP1C1 and other transporters. Then, T3 and T4 are transported to the intracellular medium by MCT8 and/or OATP1C1 in different target neural cells. T4 goes into astrocytes and some amount of it goes into interneurons and pyramidal cells (more abundantly in non-human primates). T4 is converted into T3 by astrocytic deiodinase 2 (DIO2), providing additional T3 to nearby neural cells. T4 goes abundantly into neural cells as well. Both T4 and T3 can be deiodinated to rT3 and T2, respectively, by DIO3.

This is a further step in the knowledge of TH mechanisms of control of the efferent motor system and the possible targets to look at when studying the physiopathogenics of TH transporter deficient syndromes.

### 3.6. OATP1C1 in Corpora amylacea

*Corpora amylacea* are glycoprotein-containing spherical vesicles, probably derived from astrocytes [59], with a high polysaccharide content that can be marked by Con A. They contain many components derived from astrocyte, neuron and oligodendrocyte breakdown products, such as neurofilaments and IgMs [62,63,64,65,111,112,113,114,115] as well as aggregated lipid membrane fragments and disrupted cellular organelles [116]. Their origins from both glia and/or neuron remain open and have been enunciated by various authors [60,63,117,118,119,120,121]. There is much evidence to support that *Corpora amylacea* in the human brain work as containers that cumulate waste products and engage in a brain cleaning mechanism, as these vesicles tend to drain in the meningeal lymphatic system [59,122]. The presence of *Corpora amylacea* has been associated with aging and several neurodegenerative diseases, Alzheimer’s disease [64,113,121,123,124,125], Parkinson’s disease [126,127], Huntington’s disease [123], multiple sclerosis [63,128], amyotrophic lateral sclerosis [128] and epilepsy associated with hippocampal sclerosis [118,129,130,131,132,133].

In this study, we unexpectedly found that human subpial layer I *Corpora amylacea* in the motor cortex can be stained by several OATP1C1 antibodies against different protein epitopes. All our results strongly indicate that the entire OATP1C1 protein, or a good part of it, is found inside *Corpora amylacea*. We found OATP1C1-positive *Corpora amylacea* not only in brain tissue from aged donors, but also in all tissues from younger donors (29, 32, 54 and 59 years old), as has also been documented in Cavanagh (1999) [117], who found these structures even in normal children. There are no references describing *Corpora amylacea* in young or adult monkeys. Suzuki et al. (1980) [134] found *Corpora amylacea* in two very aged baboons and never in the sites where *Corpora amylacea* are most common in humans. Cavanagh (1999) [117] reported studies in just one very old Rhesus monkey and found just a few of these vesicles in striatum and subpial feltwork. In other non-human species, the results were always negative.

The functional explanation of this is quite intriguing. As shown here, OATP1C1 is a transmembrane transporter protein abundantly expressed in astrocytes and neurons. One possibility is that the OAPT1C1 present in *Corpora amylacea* originates from damaged neural cell fragments. However, a recent paper provides another clue, reporting that the TH carrier protein transthyretin (TTR), which is the major T4 carrier in CSF and is expressed mainly in the choroid plexus and leptomeningeal epithelium [135,136,137], seems to be present in *Corpora amylacea* in hereditary transthyretin amyloidosis [138]. The expression of both T4 carrier TTR or T4 transporter OATP1C1 in *Corpora amylacea* might support a storage role of T4 inside those vesicles.

### 3.7. Modeling the Etiopathogenics of TH Transporter Deficient Syndromes

**Figure 10:**
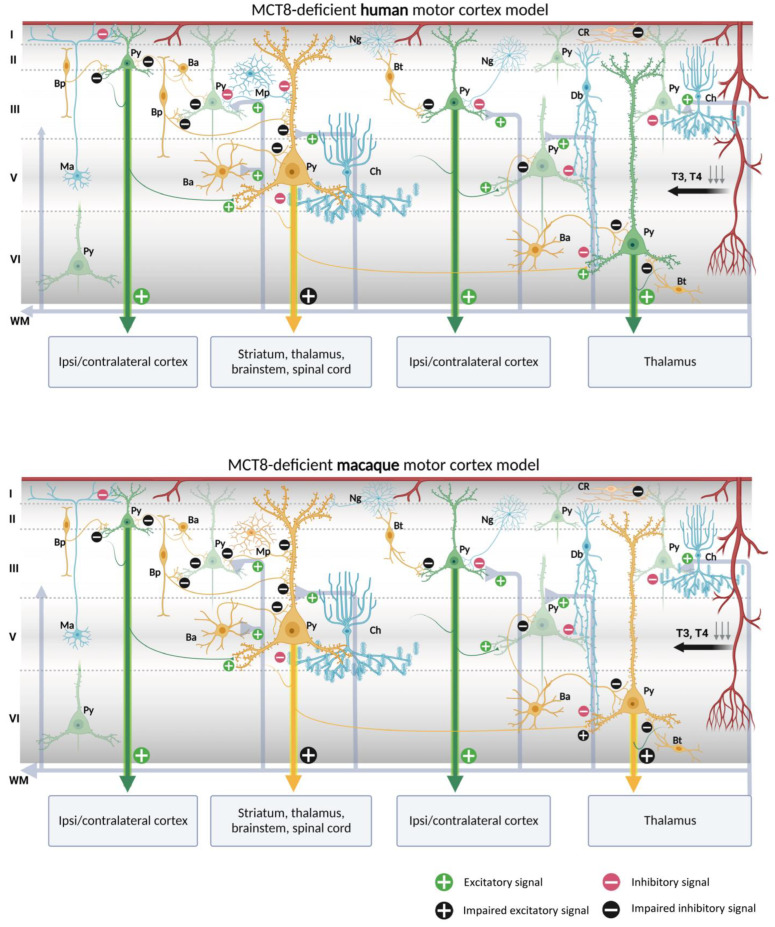
Models of human and macaque MCT8-deficient motor cortex. The models include our results and show possible cortical functional microcircuitry physiopathogenic mechanisms. A deficiency of MCT8, the most important route for circulating TH into the brain, results in a lack of TH throughout the brain. Pyramidal cells and interneurons expressing MCT8 are shown in yellow. Pyramidal cells non-expressing MCT8 are shown in green and interneurons non-expressing MCT8 are shown in blue. The lack of T3 and T4 may cause trophic deficits in pyramidal cells and interneurons, generating hyperexcitation on pyramidal cells due to the possible abnormal inhibitory function of the interneurons. Abnormalities in pyramidal cells may cause irregularities in their excitatory output signaling to intrinsic and extrinsic targets, producing symptoms such as spasticity, hypertonia, hyperreflexia, epilepsy, and others, depending on the final dendritic activity. Ba: basket cell, Bp: bipolar cell, Bt: *bitufted* cell, Ch: *chandelier* cell, CR, Cajal–Retzius cell, Db: *double bouquet* cell, Ma: *Martinotti* cell, Mp: multipolar cell, Ng: neurogliaform cell, Py: pyramidal cell, L I–VI: layers of the cerebral cortex, WM: white matter (scheme based on Szentágothai, 1975 [139] and Jones, 1993 [140]; created with BioRender.com).

**Figure 11:**
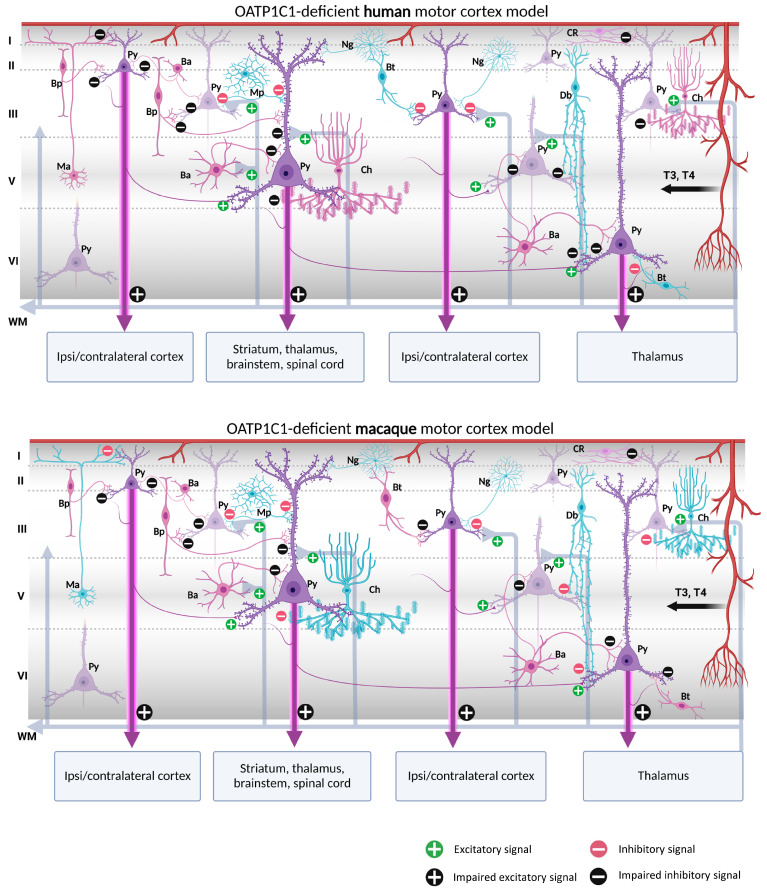
Models of human and macaque OATP1C1-deficient motor cortices. The models include our results and show possible cortical functional microcircuitry physiopathogenic mechanisms. The models might explain some of the symptoms generated in OATP1C1-deficient syndromes. Pyramidal cells and interneurons expressing OATP1C1 are shown in purple. Interneurons non-expressing OATP1C1 are shown in blue. The lack of T4 (and thus decreased deiodination by the astrocytes) may cause trophic deficits in pyramidal cells and interneurons, generating hyperexcitation on pyramidal cells due to the possible abnormal inhibitory function of the interneurons. Abnormalities in pyramidal cells may cause irregularities in their excitatory output signaling to intrinsic and extrinsic targets, producing symptoms such as spasticity, hypertonia, hyperreflexia, epilepsy and others, depending, for example, on the final dendritic activity. Ba: basket cell, Bp: bipolar cell, Bt: *bitufted* cell, Ch: *chandelier* cell, CR, Cajal–Retzius cell, Db: *double bouquet* cell, Mp: multipolar cell, Ma: *Martinotti* cell, Ng: neurogliaform cell, Py: pyramidal cell, L I–VI: layers of the cerebral cortex, WM: white matter. (Scheme based on Szentágothai, 1975 [139] and Jones, 1993 [140]; created with BioRender.com).

Our contribution on the specific cellular distribution of MCT8 and OATP1C1 transporters in the distinct subtypes of neurons and vascular elements in the motor cortex of humans and macaques raises the possibility of a modeling exercise to hypothesize what would happen in the cortical circuitry in the case of TH transport deficiency. We have summarized our reflections in Figure 10 and Figure 11.

Putting together our description of the abundance of both MCT8 and OATP1C1 in pyramidal cells of all motor cortex layers, which are the origin of the long cortico-descending, cortico-cortical and cortico-thalamic pathways, and several types of GABAergic interneurons, with all the previous knowledge on the numerous trophic actions of TH in those neurons [141,142,143,144], it can be discussed whether the lack of TH would result in poorly developed pyramidal neurons, and thus would create a deficit in the outcome of their axonal response, based mainly in poorly developed spinal dendrites, the lack of gene regulation of plasticity involved proteins, and even in the structural constituents of their long projection axons. All that together could underlie a part of the pathological symptoms that arise in TH transporter deficient syndromes.

In the normal cortical microcircuitry, the dynamics of the motor cortex are very much dependent on cell-type-specific interaction. The motor cortex receives excitatory thalamo-cortical inputs in layers III/V as well as cortico-cortical excitatory inputs in layers II/III. These inputs make synaptic contact with the pyramidal neuron basal dendrites. At the same time, the extrinsic input-induced excitation in pyramidal neurons is modulated and regulated by various types of inhibitory interneurons at different levels of the pyramidal neuron-like soma, axon, and dendritic trees, thereby maintaining the cell′s intrinsic excitation–inhibition balance and the generation of the final output action potential. As we outline in Figure 10 and Figure 11, when there is a lack of TH due to a deficiency of the MCT8 and OATP1C1 transporters, the pyramidal neurons are no longer in the same healthy synaptic state as in a normal situation, so their output is very plausibly altered, producing the typical symptoms of upper motor neuron disease, which are present in AHDS patients as bilateral Babinski reflexes and hyperreflexia [90], and, in the patient with OATP1C1 deficiency, spasticity of the lower limbs and myoclonic-like movements [24]. We cannot, so far, use these models to explain differences in the clinical aspects and phenotypes of the patients. Although there are several descriptions of patients with AHDS, there is only one description of a patient with a partial mutation of SLCO1C1, the gene coding for OATP1C1. The description is a 15.5 years old girl with normal development in the first year of life who gradually developed dementia, with spasticity and intolerance to cold [24]. Brain imaging demonstrated gray and white matter degeneration and severe glucose hypometabolism. This is quite severe a condition, to which the functional consequences of structural alterations due to the lack of TH through development should be added. We can only contribute to the knowledge on this disease showing that the insufficiency of TH transporter causes a deep alteration in cortical equilibrium excitation/inhibition.

Furthermore, in the deficiency of transporter syndromes, all inhibitions at the basal dendrites, soma, or apical dendrites in the pyramidal neurons would be altered or diminished due to a deficit in the functionality of GABAergic interneurons. This would not only increase the excitability of the pyramidal neuron, which has few resources to regulate its excitatory output to cortico-spinal, cortico-cortical, or cortico-thalamic extrinsic circuits, but also would disrupt the activity of the total cortical columns through intraneuronal relationships. As a result, motor disturbance symptoms such as epilepsy, a hallmark of TH transporter deficient syndromes, may occur. Similarly, the poor synaptic health of pyramidal neurons will impede the synaptic plasticity processes required for sensory and motor learning. Finally, changes in activity in layer I would alter the selection of motor stimuli for correct movement execution. These would explain some of the severe clinical features of the motor system in TH transporter deficiency syndromes, but it should be noted that other trophic and systemic factors occurring during development, particularly those that depend on the disease impact on the blood–brain barrier, complicate the final etiopathogenic interpretation.

One important issue to take into account is that these models refer to the effects of deficiency of TH transporters in the excitation–inhibition equilibrium of normal adult cellular architecture, and that we are aware that the many structural alterations known to appear through development in the cortex of patients with insufficient TH transporters probably would add more dysfunctional factors to the neurological syndrome.

In summary, our findings suggest that a lack of these transporters in neurons and cellular elements would significantly impact the cortex′s excitation–inhibition balance, leading to clinically severe movement impairment. For this reason, it is fundamental to know the spatial and temporal pattern of TH transporter expression in the human brain, as TH transporter localization can control the distribution of TH in the CNS and the access of TH to specific neural cells.

## 4. Materials and Methods

### 4.1. Human Samples and Tissue Preparation

The present observations are based on the analysis of postmortem material obtained from 7 normal individuals, ranging between 29 and 98 years, with no clinical or pathological evidence of neurological or psychiatric disorders (Table 1). The material was kindly provided by Dr. Ricardo Insausti, Human Neuroanatomy Laboratory, School of Medicine, University of Castilla–La Mancha (Albacete, Spain), and Dr. Lucía Prensa, Department of Anatomy, Histology and Neuroscience, Medical School, Autonomous University of Madrid (Madrid, Spain).

All human brain tissue studied for this paper was prepared with the same special protocol for neuroscience studies. The data of the length of admission in hospital or intensive care unit were not reflected in the death certificate at the time, but all the brains arrived at the necropsy laboratory between two- and twenty-four h postmortem. The necropsy was performed by a pathologist and a neurologist, and careful macroscopical and microscopical examination of samples of brain tissue was performed by the pathologist to discard edema, hemorrhages, infections, or metastasis and ensure that the brain was normal and could be used as a control sample. Tissue from donors of the same list and protocol has been used in other published papers such as Iglesias et al. (2018) [145], and Uroz et al. (2004) [146]. The tissue preparation has been extensively reported elsewhere [145,146,147,148]. Briefly, the brains were cut into thin blocks that were fixed by immersion in 4% paraformaldehyde for 10 days or 10% buffered formalin for at least 4 weeks. Blocks of brains were immersed in 15% sucrose at 4 °C until they sunk before cutting. Samples were cut with a freezing microtome into 50 μm thick sections that were serially collected in a cryoprotective solution and stored at −80 °C.

### 4.2. Monkey Samples and Tissue Preparation

In this study, we used tissue from 10 adult normal monkeys (Table 2) that had been kept in cryoprotection in Dr. Rausell′s tissue bank in the Department of Anatomy, Histology, and Neuroscience, Medical School, Autonomous University of Madrid (Madrid, Spain).

The tissue preparation has been described in detail in previous studies [49]. Briefly, monkeys were anesthetized with intramuscular ketamine and given an overdose of intravenous Nembutal. They were then perfused through the ascending aorta with normal saline followed by a solution of 4% paraformaldehyde and 1% glutaraldehyde in phosphate buffer (PB, 0.1 M, pH 7.4). The brain was removed and blocked. All the blocks were subsequently postfixed in 4% paraformaldehyde for 4 h, infiltrated with 30% sucrose in 0.1 M PB at 4 °C with gentle agitation, frozen in dry ice, and stored at −80 °C. The frozen blocks of the flattened cortex were cut tangential to the pia mater into 25–30 μm thick sections in a freezing sliding microtome. Alternate series of sections were collected in a sterile cryoprotectant solution. These series would later be processed for Nissl staining, immunohistochemistry and double immunofluorescence for MCT8/OATP1C1 and a number of cell/vessel markers.

### 4.3. Immunohistochemistry

For immunohistochemical procedures, epitopes were unmasked by heating the tissue in sodium citrate buffer (pH 6.0) or Tris–EDTA buffer (pH 8.0) at 90 °C (human tissue) or 37 °C (macaque tissue) for 30 min in some of the experiments. The activity of endogenous peroxidases was quenched by adding 10% H_2_O_2_ and 10% methanol for 15 min for 2 times. Sections were then immersed for 60 min in a blocking solution containing 5% normal serum from the species where the secondary antiserum was raised, 4% bovine serum albumin (BSA), and 0.1 M Lysine in 0.1 M PB. The sections were incubated in a solution containing primary antibodies (Table S2) diluted in 0.1 M PB containing 1% normal serum and 4% BSA for 48 h at 4 °C on a shaking platform and then in a solution containing a biotinylated secondary antibody (Table S2), specific to the primary antibody species, diluted in 0.1 M PB containing 1% normal serum and 4% BSA for 1 h at room temperature. The specific goat anti-rabbit IgG γ1 chain biotinylated secondary antibody was used when the rabbit anti-OATP1C1 was used as the primary antibody (Table S2). For signal amplification, we mainly used the avidin–biotin complex (Invitrogen, 32050) for 60 min and then revealed with 0.5 mg/mL 3,3′–5,5′-diaminobenzidine tetrahydrochloride (Sigma, D5905) and 0.01% H_2_O_2_ in 0.1 M PB or diaminobenzidine–glucose oxidase with nickel enhancement [149]. The reaction was stopped with 0.1 M cold PB and the sections were washed, mounted on gelatin-coated slides, and dehydrated in a graded series of ethanol to xylene. The slides were coverslipped with DPX.

### 4.4. Double Immunofluorescence

For double immunofluorescence staining, endogenous autofluorescence bleaching by photobleaching pre-treatment of the tissue was applied for 48 h before moving on to the other steps. Tissue antigenicity was unmasked as described above. The sections were immersed in blocking solution (5% normal serum, 4% BSA, and 0.1 M Lysine in 0.1 M PB) for 1 h at 4 °C with gentle shaking. Subsequently, sections were incubated for 72 h at 4 °C with primary antibodies (Table S2) and 3 h with fluorescent-dye-conjugated antibodies (Table S2) specific to the primary antibody species at 4 °C. Lastly, the sections were counterstained with 0.01% 4′, 6-diamidino-2-phenylindole (DAPI; Invitrogen, D1306) for 5 min and coverslipped with FluorSave (Merck, 345789). Different populations of GABAergic neurons were labeled with specific antibodies for CALB [50,51,52], CALR [50,51], nNOS [53], PARV [50,51,52] and SOM [51]. Different populations of glial cells were labeled with specific antibodies for GFAP [55], MBP [56] and IBA1 [57].

Endothelial cells were visualized directly using a DyLight 594-conjugated lectin UEA-I [150]. The lectin from UEA-I was used in conjunction with other primary antibodies to label blood vessels in free-floating sections.

### 4.5. Multiple Labeling of OATP1C1 and Markers of Corpora Amylacea

*Corpora amylacea* can be stained by Con A due to their polysaccharide properties [62,151] and present specific epitopes that can be identified by IgM class natural antibodies [61]. To eliminate the possibility of false positives caused by IgM contaminants in commercial antibodies, which had previously been reported [61], we used secondary antibodies against the γ1 chain C region of the primary rabbit IgG to recognize specifically the primary anti-OATP1C1 antibodies. Sections were incubated for 72 h at 4 °C with OATP1C1 antibodies (Table S2) and human IgM and for 3 h with fluorescein-conjugated secondary antibody at 4 °C. Then, *Corpora amylacea* were visualized directly using a Rhodamine-conjugated Con A for 12 h incubation at 4 °C. The sections were immediately washed, counterstained with 0.01% DAPI for 5 min, and coverslipped with FluorSave. We also used Congo Red staining for amyloid deposits to stain *Corpora amylacea* [152].

### 4.6. Image Acquisition and Processing

Brightfield images for stained sections were obtained assisted by computer graphics software Neurolucida (MicroBrightField Bioscience, Williston, VT, USA) that interfaced with a CX9000 digital camera attached to the microscope (Nikon Eclipse 400, Nikon Instech Co., Ltd., Kawasaki, Japan). Immunofluorescent images were captured with a confocal microscope (Zeiss, Spectral Confocal Microscope LSM710, Oberkochen, Germany) coupled with Zen 3.1 pro software. The control or reference images were acquired at the same time and under the same conditions as the respective set of images.

Image analysis and treatment were carried out using FIJI software (National Institutes of Health, USA) for the merging of images from different fluorescence channels, as well as the modification of contrast and brightness and maximum intensity projection to improve their visualization, which was processed in the same manner as the images corresponding to their respective controls.

## Figures and Tables

**Table 1:** Clinical data of the human donors.

Cases	Sex	Age (y)	Postmortem Interval (h)	Brain Weight (g)	Cause of Death
1	Male	97	9	1238	Septic shock
2	Female	98	6	1168	-
3	Male	59	<24	1020	Pneumonia
4	Male	86	<24	-	-
5	Male	29	4	1500	Lung tumor
6	Male	32	3	1420	Hemorrhagic gastroenteritis
7	Male	54	12	1350	Aortic aneurysm

**Table 2:** Data of monkey brain tissue.

Cases	Species	Age (y)	Sex
1	*M. fascicularis*	3	Female
2	*M. fascicularis*	5	Male
3	*M. fascicularis*	5	Male
4	*M. fascicularis*	5	Female
5	*M. fascicularis*	5	Male
6	*M. fascicularis*	5	Male
7	*M. fascicularis*	5	Female
8	*Squirrel monkey*	3	Female
9	*M. fascicularis*	5	Male
10	*M. mulatta*	7	Male

## Data Availability

Not applicable.

## Supplementary Materials

The following supporting information can be downloaded at: https://www.mdpi.com/article/10.3390/ijms24043207/s1.
