# Supplementary material for: Thyroid Hormone Transporters MCT8 and OATP1C1 Are Expressed in Pyramidal Neurons and Interneurons in the Adult Motor Cortex of Human and Macaque Brain
Source: Int J Mol Sci. 2023 Feb 6;24(4):3207. doi: 10.3390/ijms24043207 (PMC9965431; doi:10.3390/ijms24043207)
Supplement: Supplementary file 1 [file ijms-24-03207-s001.zip › ijms-2057397-supplementary/Wang et al. Supplementary Figures and Tables-Final_03022023-2.pdf]

Article

Thyroid Hormone Transporters MCT8 and OATP1C1 Are Expressed in Pyramidal Neurons and Interneurons in the Adult Motor Cortex of Human and Macaque Brain

Yu Wang [1,2,†], Ting Wang [1,2,†], Ana Montero-Pedrazuela [3], Ana Guadaño-Ferraz [3,*] and Estrella Rausell [1,*]

1 School of Medicine, Department of Anatomy Histology & Neuroscience, Autónoma de Madrid University (UAM), 28029 Madrid, Spain
2 PhD Program in Neuroscience, Autónoma de Madrid University (UAM)-Cajal Institute, 28029 Madrid, Spain
3 Instituto de Investigaciones Biomédicas Alberto Sols, Consejo Superior de Investigaciones Científicas (CSIC)-Autónoma de Madrid University (UAM), 28029 Madrid, Spain
* Correspondence: aguadano@iib.uam.es (A.G.-F.); estrella.rausell@uam.es (E.R.)
† These authors contributed equally to this work.

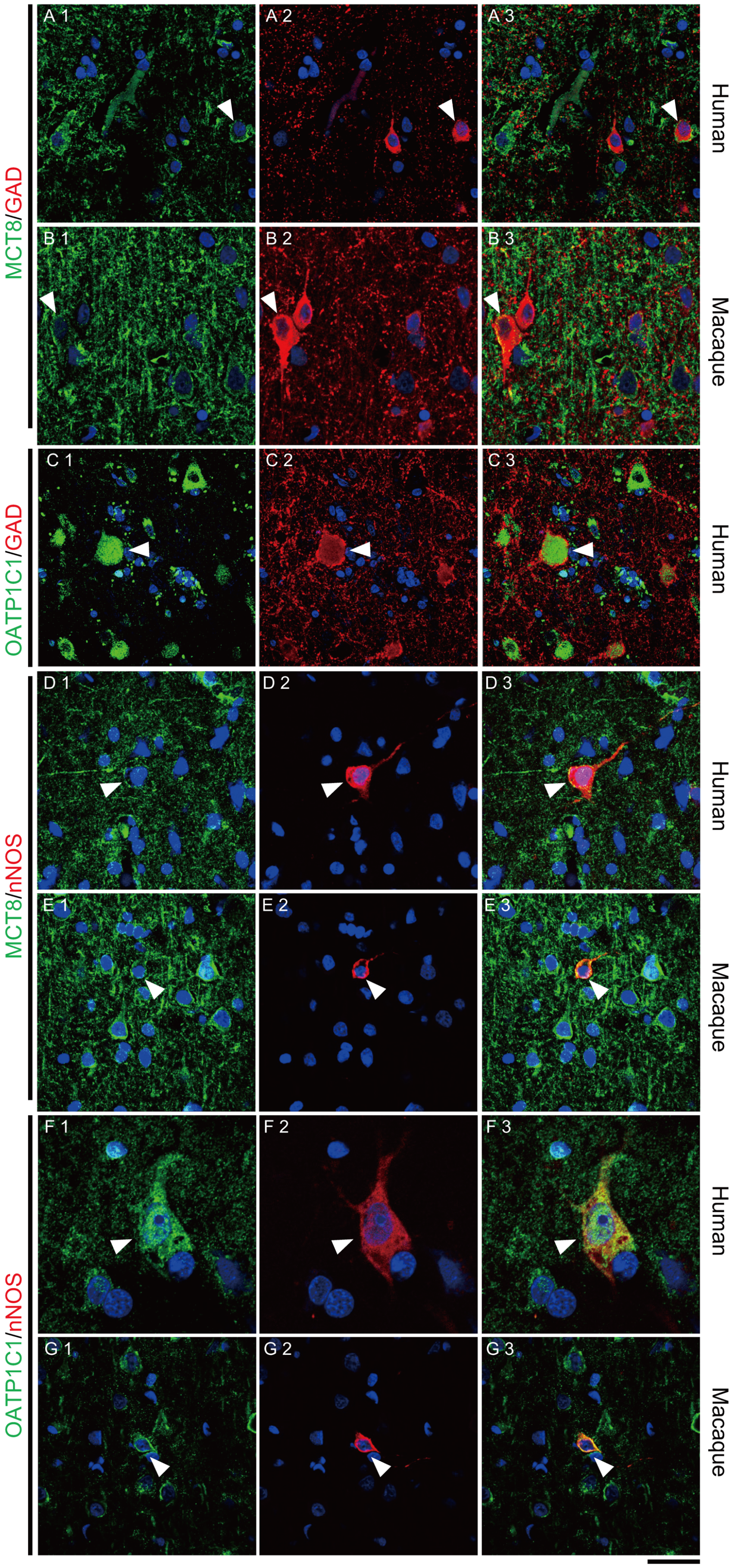

A 1
A 2
A 3
B 1
B 2
B 3
C 1
C 2
C 3
D 1
D 2
D 3
E 1
E 2
E 3
F 1
F 2
F 3
G 1
G 2
G 3
MCT8/GAD
OATP1C1/GAD
MCT8/nNOS
OATP1C1/nNOS
Human
Macaque
Human
Human
Macaque
Human
Macaque

Figure S1. Expression of MCT8 and OATP1C1 in human and macaque cortical GABAergic interneurons. Representative fluorescence confocal microscope photomicrographs showing immunostaining for MCT8 (green, **A1-B3, D1-E3**) and OATP1C1 (green, **C1-C3 and F1-G3**) in GABAergic interneurons of human and macaque motor cortex. (**A1-A3**) (human) and (**B1-B3**) (macaque) show colocalization of MCT8 (green) with the GABAergic interneuron marker GAD (red). (**C1-C3**) show colocalization of OATP1C1 (green) with the GABAergic interneuron marker GAD (red) in the human motor cortex. (**D1-G3**) show MCT8 (green, **D1-E3**) and OATP1C1 (green, **F1-G3**) colocalization with the nitrergic interneuron marker nNOS (red) in human (**D1-D3 and F1-F3**) and macaque (**E1-E3 and G1-G3**) motor cortex. Counterstaining with DAPI (blue) shows nuclei of all cells. White arrowheads point to double-stained cells. GAD: Glutamic acid decarboxylase, nNOS: Neuronal nitric oxide synthase. Scale bar = 38 μm (A1-A3), 25 μm (B1-B3 and F1-F3), and 50 μm (C1-E3 and G1-G3).

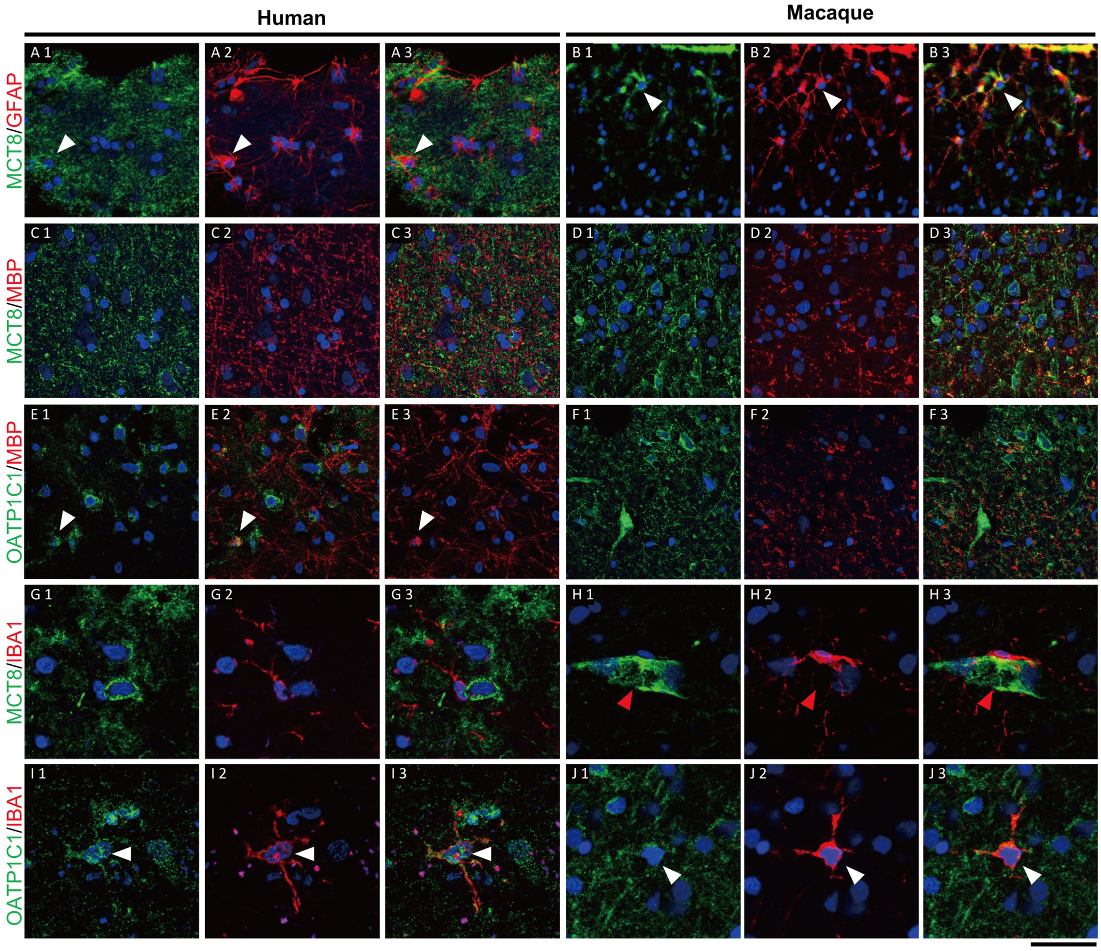

Figure S2. Expression of MCT8 and OATP1C1 in glial cells of human and macaque motor cortex. Representative fluorescence confocal microscope photomicrographs showing immunostaining for MCT8 (**A1-D3 and G1-H3**) and OATP1C1 (**E1-F3 and I1-J3**) in different glial cells of human (**A1-A3, C1-C3, E1-E3, G1-G3 and I1-I3**) and macaque (**B1-B3, D1-D3, F1-F3, H1-H3 and J1-J3**) motor cortex. (**A1-B3**) show MCT8 (green) colocalization with the astrocytes marker GFAP (red). (**C1-D3**) show some MCT8 (green) immunopositive fibers colocalize with the oligodendrocyte marker MBP (red). (**E1-F3**) show weak OATP1C1 (green) immunoreactivity colocalizes with MBP (red) in the soma of oligodendrocyte in the human motor cortex (**E1-E3**), and in some OATP1C1 immunopositive fibers in the macaque motor cortex (**F1-F3**). (**G1-H3**) show no colocalization of MCT8 (green) and the microglial cell marker IBA1 (red). (**I1-J3**) show OATP1C1 (green) colocalization with IBA1 (red). Counterstaining with DAPI (blue) shows nuclei of all cells. White arrowheads point to double stained cells. Red arrowheads point to a blood vessel. GFAP: Glial fibrillary acidic protein, IBA1: ionized calcium binding adaptor molecule 1, and MBP: myelin basic protein. Scale bar = 75 μm (A1-B3), 50 μm (C1-F3), and 25 μm (G1-J3).

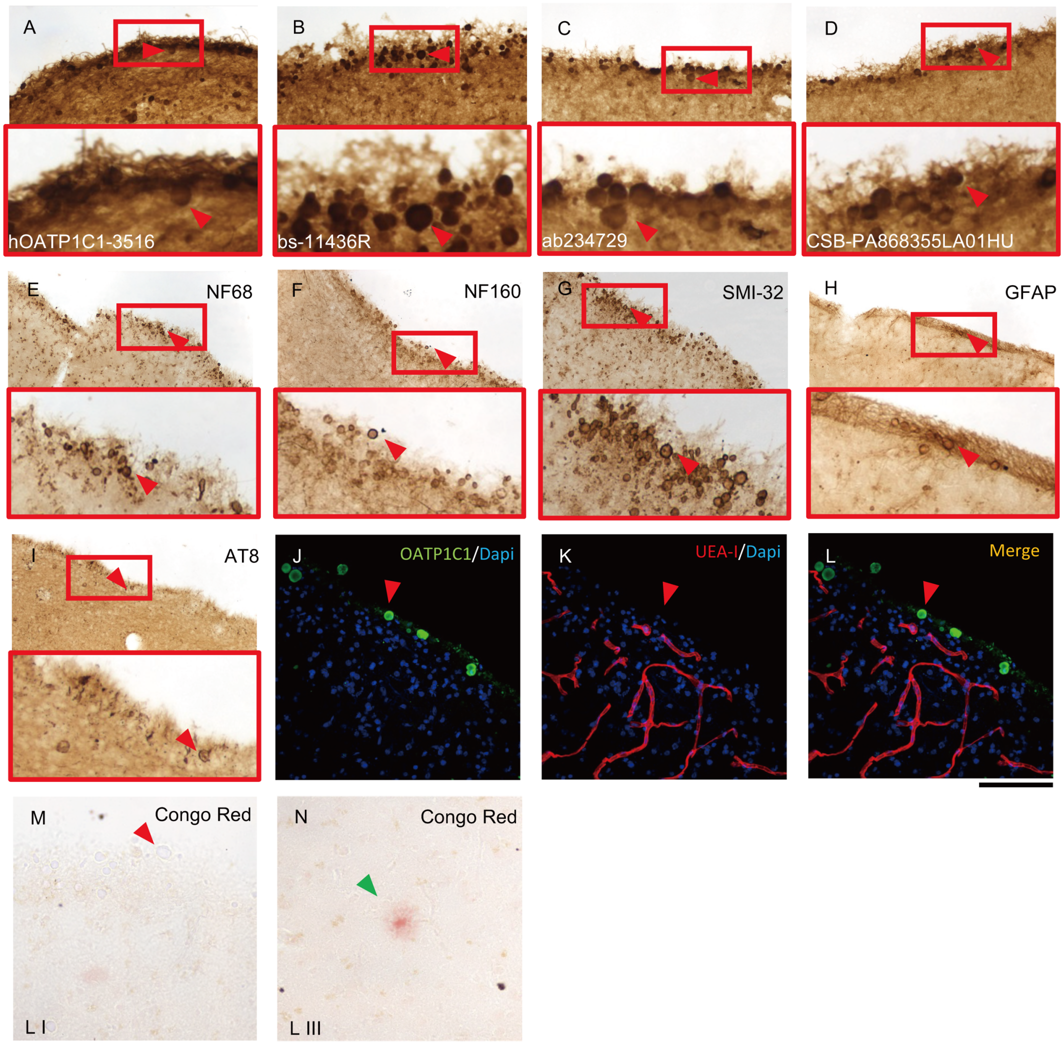

Figure S3. Immunohistochemical characterization of the *Corpora amylacea* in the human motor cortex. Representative brightfield photomicrographs (**A-I, M and N**) and immunofluorescence-labeled confocal images (**J-L**) of layers I and III of the human motor cortex. (**A-D**) A large number of aggregated spherical vesicles (red arrowheads) are visible after immunohistochemistry with different OATP1C1 antibodies (see references in insets), as well as with NF68 (**E**), NF160 (**F**), SMI-32 (**G**), GFAP (**H**), and AT8 (**I**). The regions in the red frames are enlarged below the corresponding photo. (**J-L**) Confocal microscope images double stained for OATP1C1 (green) and UEA-I (red). The red arrowheads point to OATP1C1 immunopositive vesicles that are not stained with UEA-I. Of note, since *Corpora amylacea* OATP1C1 immunoreactivity appears with much higher fluorescence excitation intensity compared with other cells, neural OATP1C1 expression is not captured in the presented images. Brightfield photomicrographs (**M and N**) for Congo Red staining of the same section in which an amyloid plaque (green arrowhead, **N**) is observed in layer III, as a positive control, but the vesicles are not stained (red arrowhead, **M**). AT8: Phospho-Tau (Ser202, Thr205), GFAP: Glial fibrillary acidic protein, NF68: Neurofilament F68, NF160: Neurofilament F160, UEA-I: Ulex Europaeus Agglutinin-I, L I-III: layers of the cerebral cortex. Scale bar = 100 μm (A-D), 33 μm (red panel in A-D), 200 μm (E-I), 66 μm (red panel in E-I), 80 μm (F-H) and 50 μm (M and N).

Video S1. Confocal microscopy 3D reconstruction of OATP1C1 localization in *Corpora amylacea*. The video shows the confocal microscopy 3D reconstruction of *Corpora amylacea* in the human motor cortex immunostained with OATP1C1 (green) and IgM (purple). Counterstaining with DAPI (blue) shows nuclei of all cells. *Corpora amylacea* contain a central compact core with a high presence of OATP1C1 and a peripheral region with IgM positive immunoreactivity.

Table S1. Summary of previous studies of MCT8 and OATP1C1 expression in the brain of different mammalian species.

Cell type	TH Transporters	Species	Distribution	Methods	References
Endothelial cells	MCT8	Fetal, juvenile and adult mouse and rat	Whole brain, thalamus, cortex, hippocampus, striatum, hypothalamus	IF, ISH, RNA-Seq	[26-30]
		Neonate to adult Cynomolgus monkey	Isolated brain microvessels	LC–MS/MS	[31]
		Fetal and adult human	Brain (different fore-mid-hind brain regions)	IF, IHC, RNA-Seq	[26,29,32-36,39,40]
	OATP1C1	Fetal and juvenile mouse, adult mouse and adult rat	Whole brain, cortex, isolated brain capillaries	IF, IHC, WB, RNA-Seq	[28-30,41]
		Adult human	Temporal lobe cortex	RNA-Seq	[32]
Ependymal cells	MCT8	Fetal, juvenile to adult mice, fetal rat	Choroid plexus	IF, IHC	[26,28,29,36]
		Fetal and adult human	Choroid plexus, ventricles	IF, IHC, ISH	[29,33,34,36,37,40]
	OATP1C1	Adult mouse and rat	Choroid plexus	IF, WB, ISH	[27,29,41]
		Fetal, neonate, infant and adult human	Choroid plexus, cerebral barriers	IF, IHC	[29,34,40]
Tanycytes	MCT8	Juvenile to adult mice, adult rat	Hypothalamus	IF, IHC	[26,29,36,38]

		Fetal human	Hypothalamus	IHC	[34]
	OATP1C1	Fetal human	Third ventricle, hypothalamus	IF, IHC	[34,40]
Leptomeningeal cells	MCT8	Fetal human	Cerebral barriers	IF, IHC	[40]
	OATP1C1	Fetal human	Cerebral barriers	IHC	[40]
Neural cells	MCT8	Fetal human	Subventricular zone, ventricular zone	IHC	[40]
	OATP1C1	Fetal human	Subventricular zone, ventricular zone, intermediate zone	IHC	[40]
Neurons	MCT8	Juvenile to adult mouse and adult rat	Cortex, hippocampus, hypothalamus, cerebellum, Islands of Calleja, amygdala, olfactory tubercle,	IF, IHC, RNA-Seq	[26,30,38]
		Fetal and adult human	Cortex, cortical plate, marginal zone, hippocampus, hypothalamus	IHC, RNA-Seq	[32-34,36,37,40]
	OATP1C1	Juvenile mouse	Cortex	RNA-Seq	[30]
		Fetal, infant and adult human	Cortex, brainstem, raphe, hypothalamus	IHC, RNA-Seq	[32,34,40,57]
Pyramidal cells	MCT8	Juvenile mouse	Cortex	IHC	[36]
		Fetal human	Hippocampus	IHC	[36]
Immature neurons	OATP1C1	Fetal human	Cortex, cortical plate, subplate	IHC	[40]

Cajal-Retzius cells	MCT8	Fetal human	Cortex	IF, IHC	[40]
	OATP1C1	Fetal human	Cortex	IHC	[40]
Granule cells	MCT8	Adult mouse	Hippocampus	IHC	[36]
		Fetal human	Hippocampus, subiculum	IHC	[36,40]
Folliculostellate cells	MCT8	Adult human	Anterior pituitary	IHC	[42]
Purkinje cells	MCT8	Adult mouse	Cerebellum	IHC	[36]
		Adult human		IF	[39]
Glia	MCT8	Fetal and adult human	Hypothalamus, median raphe	IHC	[37,40]
Radial glia cells	MCT8	Fetal human	Cortex, cortical plate, hippocampus, ventricular zone, subventricular zone	IF, IHC, ISH	[40]
	OATP1C1	Fetal human	Cerebral cortex, hippocampus, cerebral barriers, intermediate zone, subventricular zone, ventricular zone, midline raphe	IF, IHC	[40]
Astrocyte	MCT8	Juvenile mouse	Cortex	RNA-Seq	[30]
		Fetal and adult human	Temporal lobe cortex, cerebral barriers	IF, IHC, RNA-Seq	[32,40]
	OATP1C1	Juvenile mouse and rat	Cortex, hippocampus, hypothalamus, striatum	IF, ISH, RNA-Seq	[27,30]

		Fetal and adult human	n.a.	RNA-Seq	[32]
Microglia	OATP1C1	Adult mouse	Cortex	RNA-Seq	[30]
Oligodendrocyte	MCT8	Adult human	Temporal lobe cortex	RNA-Seq	[32]
	OATP1C1	Adult human	Temporal lobe cortex	RNA-Seq	[32]
Oligodendrocyte precursors	MCT8	Juvenile mouse	Cortex	RNA-Seq	[30]
	OATP1C1	Juvenile mouse	Cortex	RNA-Seq	[30]
Myelinating oligodendrocytes	OATP1C1	Juvenile mouse	Cortex	RNA-Seq	[30]
Undifferentiated cells	MCT8	Fetal human	Cortex, ventricular zone, subventricular zone	IHC	[35,40]
Primary cortical neurons and interneurons	MCT8	Human and mouse origin	In vitro	IF， WB	[36]
Cortical neuron	OATP1C1	Fetal mouse	In vitro	qPCR	[36]
Vascular signal	OATP1C1	Adult mouse and rat	Thalamus, hypothalamus, striatum, rostral perifornical area, leptomeninges (veins),	IF, WB, ISH, qPCR	[27,29]
		Fetal human	Cerebral cortex parietal lobe, subarachnoid space	IF, IHC	[29,40]
Signal surrounding	OATP1C1	Fetal human	Hypothalamus, organum vasculosum laminae terminalis	IHC	[34]

blood vessels					
n.a.	MCT8	Fetal, neonate and adult mouse and rat	Brain	WB	[29,36]
		Fetal and adult human	Brain, Anterior extramural migratory stream, cortical white matter, pituitary, hypothalamus, Infundibular stalk	IHC, WB	[36,38,40,41]
n.a.	OATP1C1	Adult mouse and rat	Brain	WB, NB	[29,41]
		Human	Brain except of pons and cerebellum, cortical tissue	qPCR, NB	[19,36]

IF, immunofluorescence; **IHC**, immunohistochemistry; **ISH**, in situ hybridization; **LC-MS/MS**, liquid chromatography tandem mass spectrometry; **n.a.**, not assessed; **NB**, northern blot; **qPCR**, quantitative real time PCR; **RNA-Seq**, RNA sequencing; **WB**, western blot.

Table S2: List of primary antibodies, lectins and secondary antibodies used.

Antibody/Lectin	Host	Clonality	Catalog #	Supplier	IHC Dilution	IF Dilution
MCT8	Rabbit	Polyclonal	HPA003353	Sigma-Aldrich	1:700-1:2000	1:600
OATP1C1	Rabbit	Polyclonal	hOATP1C1-3516	Donated by Dr. T. Visser	1:800	-
OATP1C1	Rabbit	Polyclonal	bs-11436R	Bioss	1:500	1:200
OATP1C1	Rabbit	Polyclonal	CSB-PA868355LA01HU	Cusabio	1:600	1:300
OATP1C1	Rabbit	Polyclonal	ab234729	Abcam	1:500	1:300
OATP1C1	Rabbit	Polyclonal	PA5-115919	Invitrogen	1:50-1:200	-
OATP1C1	Mouse	Monoclonal	sc-398883	Santa Cruz Biotechnology	1:50	-
AT8	Mouse	Monoclonal	#MN1020	Invitrogen	1:100	-
Calbindin-D-28K	Mouse	Monoclonal	#C9848	Sigma-Aldrich	1:2000	1:1000
Calretinin	Mouse	Monoclonal	6B3	Swant	1:2000	1:1000
Con A, Rhodamine	-	-	RL-1002- 25	Vector Laboratories	-	1:250
Endoglin	Mouse	Monoclonal	sc-20072	Santa Cruz Biotechnology	1:100	1:50
GAD	Mouse	Monoclonal	sc-365180	Santa Cruz Biotechnology	-	1:50
GFAP	Mouse	Monoclonal	G3893	Sigma-Aldrich	1:400	1:200
IgM from human serum	-	-	I8260	Sigma-Aldrich	-	1:25
IBA1	Goat	Polyclonal	ab5076	Abcam	1:1500	1:800
MBP	Mouse	Monoclonal	ab24567	Abcam	1:1000	1:500
NF160	Mouse	Monoclonal	MAB5254	Sigma	1:200	-
NF68	Mouse	Monoclonal	N5139	Sigma	1:50	-
nNOS	Goat	Polyclonal	AF2416	R&D systems	15 μg/mL	10 μg/mL
Parvalbumin	Mouse	Monoclonal	#P3088	Sigma-Aldrich	1:2000	1:1000
PDGFR-β	Goat	Polyclonal	AF385	R&D systems	-	1:100
RC3/Neurogranin	Rabbit	Polyclonal	bs-11435-A594	Bioss	-	1:200
SMI-32	Mouse	Monoclonal	-	Sternberger-Meyer Immunocytochemicals	1:400	1:800
Somatostatin	Mouse	Monoclonal	sc-55565	Santa Cruz Biotechnology	1:500	1:300
UEA-I, DyLight™ 594	-	-	DL-1067-1	Vector Laboratories	-	1:50

IHC, immunohistochemistry; **IF**, immunofluorescence.

Antibody	Catalog #	Supplier	IHC Dilution	IF Dilution
Goat anti-Rabbit Biotinylated	BA-1000	Vector Laboratories	1:200	-
Horse anti-Mouse Biotinylated	BA-2000	Vector Laboratories	1:200	-
Rabbit anti-Rat Biotinylated	BA-4000	Vector Laboratories	1:200	-
Goat anti-Rabbit Ig gamma-1 chain C region Biotinylated	OASB01959	Aviva Systems Biology	1:200	-
Goat-anti-Mouse IgG (AF647)	A21236	Invitrogen	-	1:500
Goat-anti-Mouse IgG (AF546)	A11030	Invitrogen	-	1:500
Goat-anti-Rabbit IgG (AF488)	A11034	Invitrogen	-	1:500
Donkey-anti-Rabbit IgG (AF488)	A21206	Invitrogen	-	1:500
Donkey-anti-Rabbit IgG (AF647)	A31573	Invitrogen	-	1:500
Donkey-anti-Goat IgG (AF488)	A11055	Invitrogen		1:500
Donkey-anti-Goat IgG (AF546)	A11056	Invitrogen	-	1:500
Donkey-anti-Mouse IgG (AF647)	A31571	Invitrogen	-	1:500
Donkey-anti-Mouse IgG (AF546)	A10036	Invitrogen	-	1:500
Goat anti-Human IgM (AF647)	A21249	Invitrogen	-	1:200
